# Manipulating mammalian cell morphologies using chemical-mechanical polished integrated circuit chips

**DOI:** 10.1080/14686996.2017.1388135

**Published:** 2017-10-27

**Authors:** Hassan I. Moussa, Megan Logan, Geoffrey C. Siow, Darron L. Phann, Zheng Rao, Marc G. Aucoin, Ting Y. Tsui

**Affiliations:** ^a^ Department of Chemical Engineering, University of Waterloo, Waterloo, Canada; ^b^ Waterloo Institute of Nanotechnology, University of Waterloo, Waterloo, Canada

**Keywords:** Tungsten, chemical-mechanical polish, Vero cells, integrated circuit, cellular response, 30 Bio-inspired and biomedical materials, 102 Porous / Nanoporous / Nanostructured materials, 212 Surface and interfaces

## Abstract

Tungsten chemical-mechanical polished integrated circuits were used to study the alignment and immobilization of mammalian (Vero) cells. These devices consist of blanket silicon oxide thin films embedded with micro- and nano-meter scale tungsten metal line structures on the surface. The final surfaces are extremely flat and smooth across the entire substrate, with a roughness in the order of nanometers. Vero cells were deposited on the surface and allowed to adhere. Microscopy examinations revealed that cells have a strong preference to adhere to tungsten over silicon oxide surfaces with up to 99% of cells adhering to the tungsten portion of the surface. Cells self-aligned and elongated into long threads to maximize contact with isolated tungsten lines as thin as 180 nm. The orientation of the Vero cells showed sensitivity to the tungsten line geometric parameters, such as line width and spacing. Up to 93% of cells on 10 μm wide comb structures were aligned within ± 20° of the metal line axis. In contrast, only ~22% of cells incubated on 0.18 μm comb patterned tungsten lines were oriented within the same angular interval. This phenomenon is explained using a simple model describing cellular geometry as a function of pattern width and spacing, which showed that cells will rearrange their morphology to maximize their contact to the embedded tungsten. Finally, it was discovered that the materials could be reused after cleaning the surfaces, while maintaining cell alignment capability.

## Graphical abstract

**Figure:**
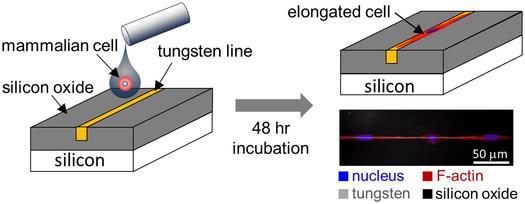

## 1. Introduction

To properly maintain organ function in humans and other mammals, cells organize in specific patterns with their extracellular matrix (ECM) and rearrange their morphology to elongate on a common axis. These cells are found in tissues such as tendons, muscles, bones, and the corneal stroma [1]. For example, smooth and skeletal muscle cells grow in the definitive axis of contraction to effectively generate the mechanical forces required by the human body [2]. The collective alignment of muscle cells allows weak forces generated by individual cells to combine and produce strong sustainable mechanical contractions. In the cornea, the corneal stroma along with fibroblast cells and the ECM are organized into patterns to maintain their functionality [3–5]. Furthermore, growth, differentiation, and cytoskeleton organization of human stem cells are influenced by ECM nanotopography [6,7]. For the past decade, there has been considerable interest in tissue engineering [1,8–10], cell adhesion on medical implants [11–13], and wound healing [14,15] that use engineered biomaterial surfaces to mimic native tissue functions. This has included synthesizing artificial scaffolds or other devices to stimulate or guide cells and ECM to form organized patterns for medical devices [1,11,16], cell immobilization [17–19], tissue engineering [8–10,20–22], and to promote tissue healing [14,15,23]. Some of these biomaterial devices consist of lithographic patterned or self-assembled surface structures [8,9], such as pillars [10,24,25], pits [10,26], grooves [6,7,24,27,28], and ribbons [29] on silicon or polymeric substrate surfaces. Substrates with printed protein patterns are also used as a platform to control cell morphology [30–32]. Common proteins used to print these devices include collagen [30] and fibronectin [31].

While progress has been made to manipulate cellular function using these devices, it is often difficult to implement these devices commercially or to reuse them after rework processes. This is partially due to the delicate nature, mechanical reliability, and defect sensitivity of the small surface features. For example, mechanical contact by small foreign particles on the surface of the device may cause permanent damage. Jahed et al. [33] demonstrated that *3T3 Swiss Albino* fibroblast cells were able to detach palladium nanopillars from the substrate, and mechanically deform nickel pillars. Even sub-micron scale *Staphylococcus aureus* bacterial cell and ECM networks can bend strong nanocrystalline nickel nanopillars [34] and poly(dimethyl siloxane) micropillars [35]. Additionally, contaminants can fall into the gaps between the small structures, and cannot be removed easily thus altering the pattern geometries and rendering the device ineffective. Hence, rework or reuse of these topographic-based devices is difficult because it is challenging to remove adherent cells or other foreign particles without damaging the patterned structures. Furthermore, high aspect-ratio soft compliant polymeric pillars or patterned lines may clump together as a result of van de Waals attractions when the distances between them are small, thereby losing their effectiveness in manipulating cells. The latter may only be improved using expensive specialty chemicals to functionalize the surface. In addition, devices with printed protein patterns are chemically fragile since the organic molecules may decompose with time and require a protective environment for long-term storage.

The primary objective of this work was to demonstrate the capabilities of a new platform of silicon-based biomaterial devices for eukaryotic cell immobilization and morphology control. These are the first devices reported in the literature that allow surface contaminants to be removed using simple chemical-free mechanical rework processes, while maintaining their functionality. Another goal was to develop a mathematical model to describe the adherent cell attachment characteristics on these devices. These materials are manufactured using integrated circuit (IC)-based tungsten chemical-mechanical polish (W-CMP) techniques [36–39] and consist of blanket silicon oxide thin films embedded with micro- and nano-meter scale tungsten on the surface. The final surfaces prepared by W-CMP techniques are hard, flat, and smooth across the entire substrate, with a root-mean-square roughness of less than 10 nm [40–42]. This is distinctively different from conventional devices that have fragile protruding structures, such as pillars or lines. Tungsten is one of the strongest and hardest metals in elemental form. Its alloys have been used to replace depleted uranium as kinetic energy penetrator ammunitions and lead, as bullet cores, to reduce the associated environmental impact. It has also been widely accepted for uses in medical neural implant sensors as chronic multi-electrodes [43–46]. Tungsten has a hardness of 14–15 GPa [47], an elasticity modulus of 410 GPa [48], and is one of the elemental metals with the highest surface energy at ~3.3 J/m^2^ [49]. Silicon oxide, which is commonly used in the IC industry, is also strong, with a hardness and elastic modulus of 8.3 GPa [50] and 69.3 GPa [48], respectively. The surface energy of silicon oxide is 0.259 ± 0.003 J/m^2^ [51]. The work of adhesion of virgin and chemical-mechanical polished silicon oxide in water is 0.06299 and 0.06304 J/m^2^ [52]. This suggests that the polishing process does not significantly alter the oxide surface chemistry. The high strength characteristics of tungsten and silicon oxide improve mechanical reliability, reduce potential mechanical contact damage, and readily allow particle defect removal from these devices, which reduces sensitivity for fall-on particle defects and allows reuse after a simple rework process. This is a well-established practice in the IC fabrication industry to remove fall-on particles, contaminant residues, and scratch defects [53,54]. Reusability will reduce the effective cost of these devices.

The new W-CMP devices utilize cell-preferential adhesion characteristics between tungsten and silicon oxide areas to attract cells to targeted locations, while the custom-designed patterns are used to control cell morphologies. No external mechanical or electromagnetic force is needed to operate this device. All of the cell migration and morphology changes are self-driven by the eukaryotic cells. To test this, a common adherent mammalian kidney epithelial cell line (Vero) was used. These cells are commonly used in virus and parasite amplification [55] and vaccine manufacturing [56,57]. Herein, Vero cells were deposited on to tungsten comb structures of various geometries. The resulting adherent cell morphologies, orientations, and proliferation were characterized. A time course study using Vero cells was performed to show the cell adhesion and spreading process on the patterned surfaces. Additional experiments with controlled biological contaminants (QT-35 quail fibrosarcoma cells) were conducted to demonstrate that cell affinity to the surfaces remains unchanged after chemical-free surface cleaning and reuse. This is believed to be the first cell morphology control platform that is capable of reuse after a chemical-free surface cleaning process. A simple mathematical simulation shows that cells reshape themselves to maximize their contact to the tungsten portions of the surface. To the best of our knowledge, this is the first mathematical model that successfully describes pattern-dependent cell alignment behavior.

## 2. Experimental

### 2.1. Integrated circuits

**Figure 1.**
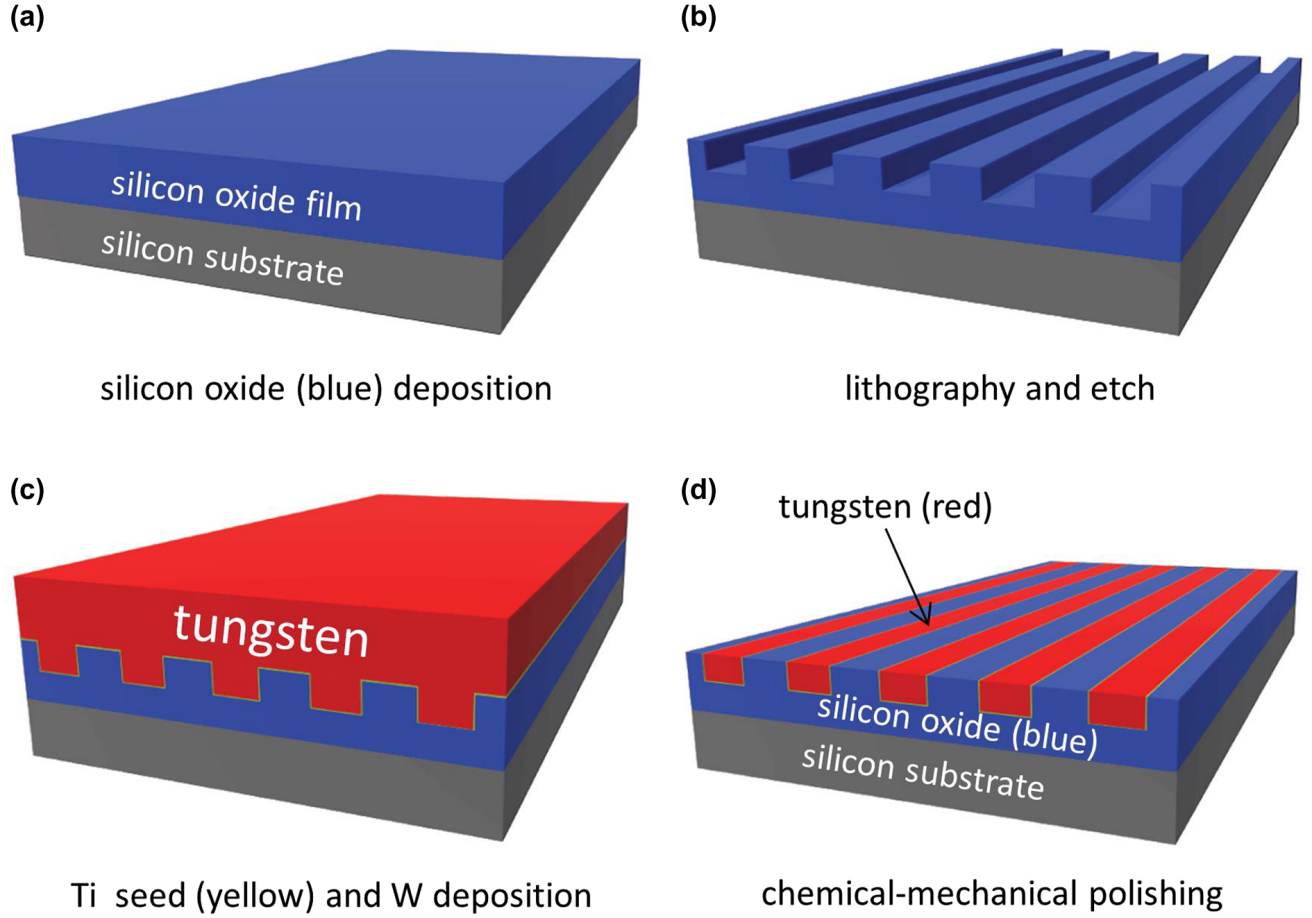
Schematic drawings depict the specimen fabrication process. The thin titanium (Ti) seed layer is shown in yellow, silicon oxide in blue, and the tungsten (W) layer in red. Note the final specimen surfaces are smooth and flat without protrusions.

Tungsten chemical-mechanical polish (W-CMP) specimens were provided by Versum Materials, LLC (Tempe, AZ, USA). The specimens were prepared using an advanced IC industrial fabrication technique on 200-mm silicon wafers [37–39]. Briefly, an overview of the fabrication process is displayed in Figure 1. Patterns were transferred to the silicon substrates coated with silicon oxide thin films using photo-lithography and plasma etching techniques, as shown in Figures 1(a) and (b). After filling the etched patterns with a thin titanium glue/seed layer (less than 20 nm) and tungsten (Figure 1(c)), the excess tungsten, titanium, and silicon oxide were removed using the W-CMP techniques [36,58–60]. The polishing step has two major components: tungsten metal oxidation, and mechanical removal of the by-products from the surface. The W-CMP slurry is commonly composed of oxidizers, such as hydrogen peroxide and ferrite nitrate [36,58–60]. The final specimen surface (Figure 1(d)) is smooth, flat, and continuous.

### 2.2. Baseline Vero cell culture and fixation processes

Vero cells (CCL-81) obtained from the American Type Culture Collection (ATCC, Manassas, VA, USA), were cultured in a 50:50 mix of Corning^®^ Cellgro^™^ Dulbecco’s Modified Eagle Media (DMEM) and F12 (Corning, NY, USA) media, supplemented with 4-mM L-glutamine (Sigma-Aldrich, St Louis, MO, USA) and Gibco^™^ 10% (v/v) fetal bovine serum (FBS) (Thermo Fisher Scientific, Waltham, MA, USA). Cells were maintained in tissue-culture treated 175 cm^2^ flasks (Corning Falcon, Corning, NY, USA) in 25 mL of media and incubated in 5% CO_2_ at 37 °C. The polished specimens were sterilized in 2 mL of 70% ethanol for 30 seconds prior to cell deposition. Specimens were air-dried, then rinsed with 2 mL of Dulbecco’s phosphate-buffered saline (D-PBS) to remove residual contaminants. Unless otherwise stated, sterilized specimens were inoculated with 0.5 × 10^5^ to 2 × 10^5^ cells/mL and incubated at 37 °C from 0.5 to 49 hours, depending on the experiment specifications. The incubation process was conducted in 6-well tissue culture plates (Nunc, Thermo Scientific, Roskilde, Denmark).

After incubation, the spent media was removed and the specimens were rinsed with 2 mL of D-PBS solution. The adherent cells on the surfaces were fixed using 2 mL of 2% methanol-free formaldehyde (Sigma-Aldrich) for 1 hour at room temperature. The cells were then permeabilized with 2 mL of 0.1% Triton-X 100 (Sigma-Aldrich) for 5 minutes. After rinsing the specimens with PBS, specimens were blocked with 2 mL of 1% (w/w) bovine serum albumin (BSA) (Sigma-Aldrich) followed by the application of deep red CytoPainter F-Actin stain (ab112127 Abcam, Cambridge, MA, USA) diluted by a factor of 1000 in 1% BSA. The sample was stained for 1 hour at room temperature in the dark. Cells were rinsed and submerged in 2 mL of 0.4 μg/mL of the 4′,6-diamidino-2-phenylindole (DAPI) (Life Technologies^™^, Carlsbad, CA, USA) for 5 minutes and then rinsed twice with 2 mL of D-PBS. Four drops of Prolong Gold anti-fade reagent (Life Technologies™, USA) were added to the final solution. Specimens were stored at 4 °C in the dark prior to imaging. Stained cells were inspected with a Leica TCS SP5 confocal fluorescence microscope (Wetzlar, Germany) at the University of Guelph, Canada. This instrument is equipped to acquire signals from five different wavelengths simultaneously. DAPI and CytoPainter F-Actin fluorescence stains were inspected with wavelengths in the ranges of 436–482 nm and 650–700 nm, respectively.

Specimens prepared for scanning electron microscopy (SEM) were fixed using 2 mL of 2% methanol-free formaldehyde for 1 hour at room temperature. Samples were dehydrated by submerging them sequentially in 2 mL of 50, 75, and 95% (v/v) ethanol solutions for 10 minutes each, followed by three 5-minute incubations using 2 mL of 100% ethanol. Specimens were dried gently with high-purity nitrogen after the final ethanol soak and stored in a nitrogen box.

### 2.3. Cell culture media prepared for protein adsorption study

The effects of cell culture media composition on cell nuclei alignment were evaluated. In this study, three media cultures were used. They include: (i) baseline control media with 10% (v/v) FBS as described in section 2.2; (ii) OptiPRO^TM^ SFM cell culture media (Life Technologies™, Thermo Fisher Scientific, Waltham, Massachusetts, USA); and (iii) OptiPRO™ media with 10% (v/v) FBS. All three media were seeded with 0.5 x 10^5^ cells/mL. Cells were incubated in their respective media for 48 hours at 37 °C, fixed with 2% paraformaldehyde, and ethanol-dried according to the procedures described in section 2.2 above.

### 2.4. Controlled cell contaminants for rework study

To demonstrate the mechanical rework capability, quail fibrosarcoma cells (cell line QT-35, European Collection of Authenticated Cell Cultures) served as the controlled adherent cell contaminants and were deposited on virgin W-CMP structures. QT-35 cells were maintained in 25 mL media of HyClone™ Minimum Essential Media (MEM) with Earle’s balanced salt solution (EBSS) (GE Health Care Life Sciences, Pittsburgh, PA, USA), 1% non-essential amino acids (NEAA) (Sigma-Aldrich), 10% (v/v) FBS, and 2 mM L-glutamine on T175 flasks (Corning Falcon) with 5% CO_2_ at 37 °C. Cells were deposited on to the W-CMP specimens in the maintenance media cell concentration of 0.5 × 10^5^ cells/mL. After 24 hours of incubation at 37 °C in 5% CO_2_, the adherent cells were fixed and ethanol-dried using the same methods described in section 2.2. Dried specimens were stored in a high-purity nitrogen environment for four days prior to the mechanical rework process. This was done to simulate typical cell alignment experiments and common SEM specimen storage practices. All QT-35 cells were subsequently removed from the device surfaces by manual scrubbing with wet cotton balls (Equate Brand, Regular Size, Walmart, Canada) in de-ionized water. This process was conducted under ambient conditions and was not in a cleanroom environment. After all adherent QT-35 cells were removed by manual scrubbing, specimens were rinsed and dried with ethanol. To evaluate the cell alignment performance following the mechanical rework processes, Vero cells were seeded on virgin and reworked W-CMP chips using the methods described in section 2.2, with an initial cell seeding density of 0.5 × 10^5^ cells/mL. Adherent cells were incubated for 48 hours, fixed, and ethanol-dried prior to SEM inspection under the conditions listed in section 2.2.

### 2.5. Quantification of cell nuclei dimension and orientation

**Figure 2.**
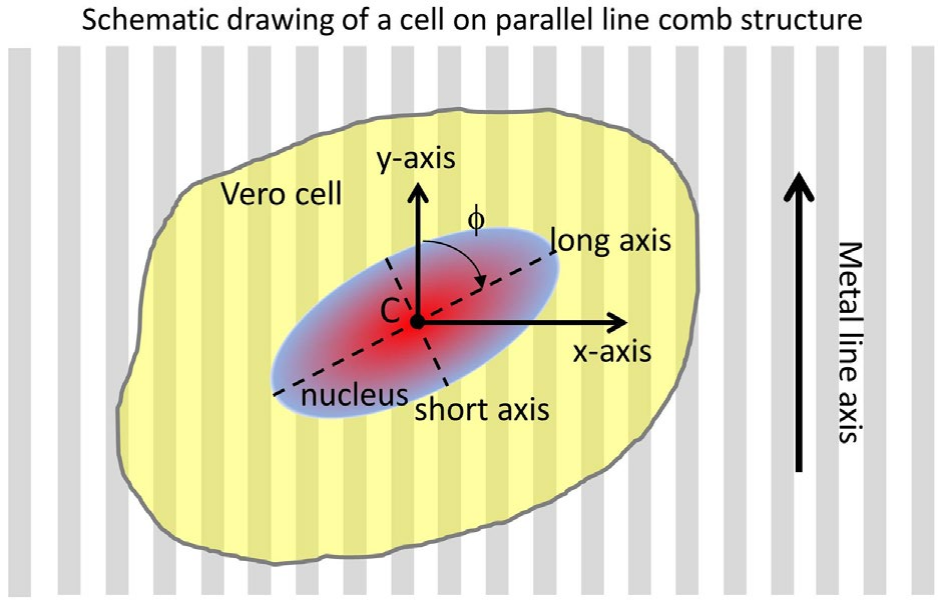
Schematic drawing of a cell on tungsten/silicon oxide patterned comb structure and their orientation parameters.

To quantify the affinity of cells for tungsten, cell nuclei orientation with respect to the patterned tungsten line axes was characterized using the angle (ϕ) that exists between the long axis of the elliptical-shaped nuclei and the metal line axis (y-axis). A schematic drawing illustrating the angle (ϕ) between these axes is shown in Figure 2. Specifically, the angle of a nucleus’ long axis that is parallel to the metal line axis (y-axis) is 0°, while the angle of a long axis that is oriented perpendicular to the metal line axis is 90°. The dimensions of cell nuclei along the long and short axis were also characterized as indicated in Figure 2. The cell nucleus center point (C) is defined as the location where these two axes intercept. These measurements were conducted manually by using the Image Processing and Analysis in Java (ImageJ) software (National Institute of Mental Health, Bethesda, Maryland, USA) built-in application tools: Angle and Straight. Data from cells located within the 50 μm region from the boundary of the comb structures were excluded in order to avoid possible edge influences.

### 2.6. Field-emission scanning electron microscopy and atomic force microscopy

A high-resolution field-emission scanning electron microscope (Zeiss 1550, Carl Zeiss AG, Oberkochen, Germany) was used for inspection and imaging. The instrument was operated with 3–7 kV of accelerated voltage with a secondary electron detector. Unless otherwise stated, no gold coating was prepared on SEM specimens. Surface roughness was measured with a Dimension 3100 Scanning Probe Microscope (Veeco, NY, USA) atomic force microscope (AFM).

## 3. Results and discussion

### 3.1. Test structure characterizations

**Figure 3.**
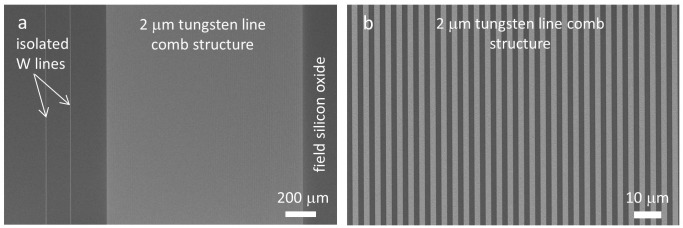
(a) SEM micrograph of a 2 μm tungsten line comb structures and two isolated tungsten lines with same width. (b) Micrograph shows a close-up image of parallel tungsten/silicon oxide line structures with line widths of 2 μm.

Typical top-down SEM micrographs of 2.0 μm isolated and dense-parallel tungsten line comb structures used in this work are shown in Figure 3(a). Isolated tungsten lines of length ~1.5 mm were embedded in silicon oxide thin films with adjacent tungsten lines at least 50 μm apart. In dense-parallel comb structures (Figure 3(b)), alternating equal-width sections of tungsten and silicon oxide are repeated. In addition to the 2.0 μm structures displayed in Figure 3, specimens with line widths of 0.18, 0.25, 0.5, 1, 2, 5, 10, 50, and 100 μm were also used in this work. In Figure 3, the SEM micrographs showed that the specimen surfaces were free of defects, such as scratches or residues. More importantly, the tungsten line sidewalls were smooth and continuous. A description of the physical dimensions of the different parallel line comb structures investigated in this work is summarized in Table 1.

**Table 1.** Data summary of tungsten/silicon oxide comb structures: physical dimensions, inspected areas, number of cells inspected, % population of cells with 10° > ϕ > −10° and 20° > ϕ > −20° of the metal line axis. The culture media initial cell concentration used was 2 × 10^5^ cells/mL. Data spreads correspond to one standard deviation.

Structure	W line width	silicon oxide line width	inspected comb structure area	number of cells in structure	cell density (cells/mm^2^)	% population aligned ±10^o^ from W lines	% population aligned ±20^o^ from W lines
	(μm)	(μm)	(mm^2^)	(n)	(cells/mm^2^)	(10^o^ > ϕ > −10^o^)	(20^o^ > ϕ > −20^o^)
1	0.18	0.18	1.8	308	171	16 ± 2	30 ± 2
2	0.25	0.25	1.8	300	167	22 ± 2	36 ± 5
3	0.5	0.5	1.8	500	278	23 ± 2	47 ± 1
4	1	1	1.8	403	224	31 ± 1	53 ± 6
5	2	2	3.6	525	146	27 ± 5	52 ± 3
6	5	5	1.8	402	223	50 ± 7	73 ± 6
7	10	10	3.6	804	223	56 ± 3	73 ± 1
8	50	50	2.0	378	189	41 ± 3	66 ± 3
9	100	100	9.0	1434	159	32 ± 1	50 ± 3
10	10	90	8.4	1184	141	87 ± 1	91 ± 1

**Figure 4.**
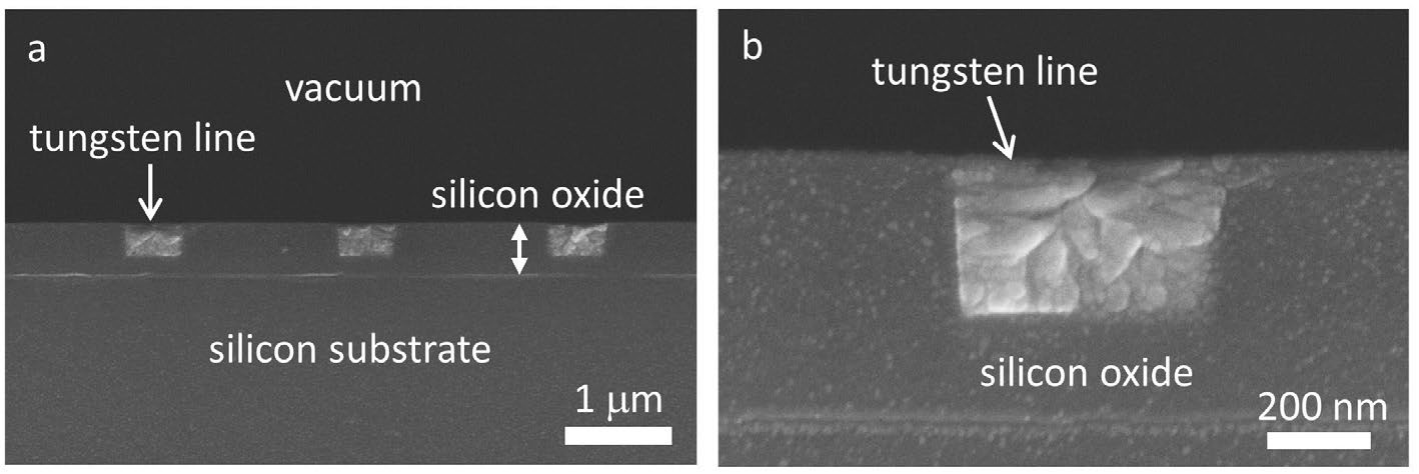
(a) Cross-sectional SEM images of dense metal patterns with 0.5 μm wide tungsten lines with 1.5 μm spacing. (b) A high magnification cross-sectional SEM image of a 0.5 μm wide tungsten line.

The surface topographic features of the tungsten line structures were characterized using cross-sectional SEM inspection and AFM techniques. A test structure with ~0.5 μm tungsten lines was cleaved perpendicular to the metal lines, and the fractured surfaces were inspected using SEM following gold coating. Typical low- and high-magnification micrographs of these cross-sectioned structures are shown in Figures 4(a) and (b). The micrographs show ~0.5 μm wide tungsten lines separated by ~1.5 μm of silicon oxide. As expected, the polished surface is smooth and flat. The silicon oxide film had a uniform thickness of ~0.5 μm and the tungsten lines were embedded to a depth of ~0.3 μm in the oxide film. Additional SEM micrographs, revealing polished blanket tungsten film and other comb structures, are displayed in Figure S1; these demonstrate the surface flatness of the specimen. It is important to note that the tungsten patterns are imbedded and anchored in the silicon oxide films. This helps to prevent these micro- and nano-meter scale patterns from damage or distortion during mechanical contacts. The root-mean-square roughness of post W-CMP silicon oxide and tungsten metal measured using AFM with scan areas of 5 μm × 5 μm were ~0.26 ± 0.06 nm and ~0.44 ± 0.14 nm, respectively. Data spreads represent one standard deviation. These roughness values are comparable with those of chemical-mechanical polished surfaces reported in the literature [42,61].

### 3.2. Vero cells on unpatterned flat surfaces

**Figure 5.**
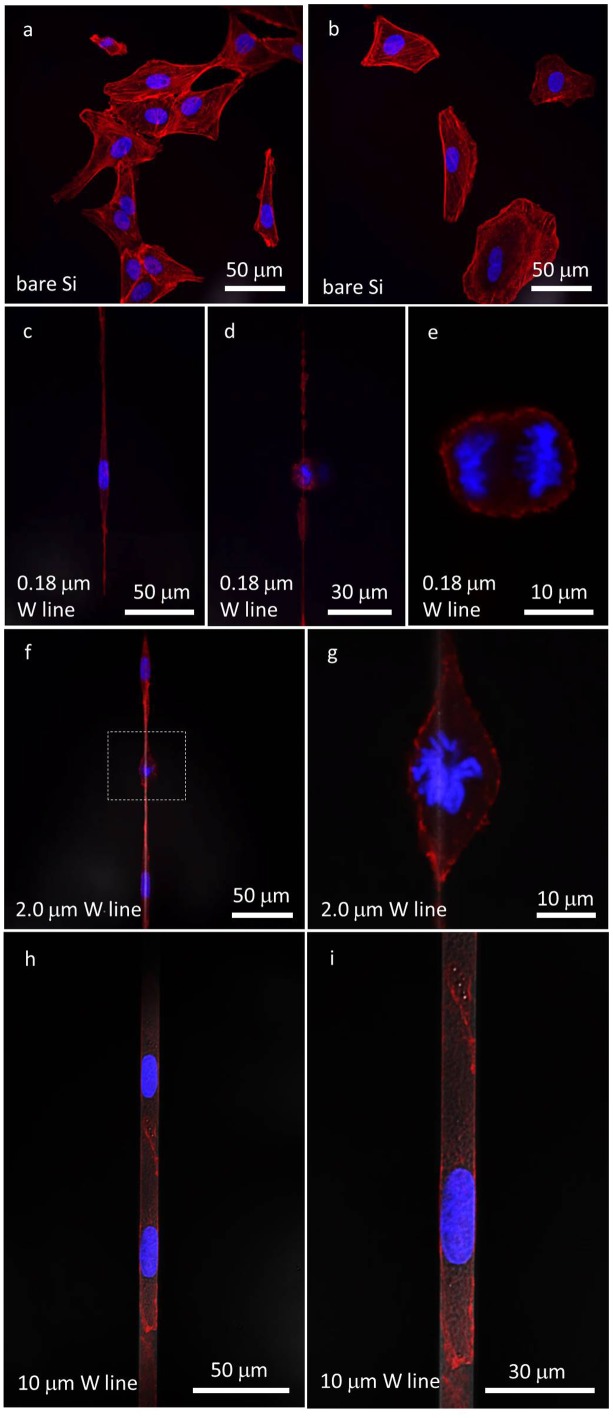
(a) and (b) Confocal fluorescence micrographs of Vero cells on the bare silicon substrate. (c) A non-dividing cell elongated on a 0.18 μm wide isolated tungsten line. (d) and (e) Micrographs of a dividing cell at low and high magnification, respectively. (f) Two non-dividing cells and one dividing cell attached to a 2 μm wide isolated tungsten line. (g) High-magnification image of a dividing nucleus. (h) and (i) Micrographs of cells attached to a 10 μm wide isolated tungsten line at low and high magnifications, respectively. Cell nuclei were labeled with DAPI and appear blue. F-actin microfilaments were stained with red fluorescent phalloidin conjugate and appear red. All cells were incubated for 24 hours.

To illustrate adherent Vero cell geometry on flat surfaces, high-resolution fluorescence confocal micrographs of Vero cells were incubated on smooth and flat bare silicon substrates for 24 hours (Figures 5 (a) and (b)). The initial cell concentration in the culture media was maintained at 2 × 10^5^ cells/mL. These silicon substrates have a native thin layer of silicon oxide at the surface as a result of exposure to atmospheric conditions. Cell nuclei labeled with DAPI (blue) and F-actin microfilaments (red) were stained with phalloidin conjugate. These micrographs reveal the morphology of Vero cells when they are in close proximity to each other (Figure 5(a)) and when they are spread apart (Figure 5(b)). Morphologies of typical adherent cells on polished field tungsten and polished field silicon oxide surfaces can be found in supplementary Figure S2. The average cell nuclear sizes on bare silicon, polished tungsten, and polished silicon oxide are summarized in Table 2. Results showed that the adherent cells on these three surfaces were randomly distributed and did not exhibit any preferential orientation, shape, or geometry. The cell nuclei appeared elliptical with the average ratio of the long and short axes of 1.4 ± 0.2, 1.7 ± 0.3 and 1.7 ± 0.4, respectively. These results demonstrated that the nuclear dimensions of Vero cells deposited on the bare silicon, polished tungsten, and polished silicon oxide surfaces are similar.

**Table 2.** Average long and short axis lengths of cell nuclei incubated on bare silicon, polished blanket tungsten, and polished silicon oxide for 24 hours. Data spreads correspond to one standard deviation.

Specimen	Number of cells inspected	Long-axis length (μm)	Short-axis length (μm)	Long-to-short axis length ratio
bare silicon	184	23 ± 5	17 ± 4	1.4 ± 0.2
polished tungsten	494	22 ± 4	13 ± 2	1.7 ± 0.3
polished silicon oxide	72	20 ± 4	12 ± 3	1.7 ± 0.4

### 3.3. Individual cell interactions with micro- and nano-meter scale isolated tungsten lines

To understand the behavior of Vero cells on micro- and nano-meter scale-wide structures, cells were incubated for 24 hours on surfaces with isolated tungsten lines of varying widths. These isolated tungsten lines were at least 1500 μm long and separated from the nearest tungsten structures by at least 50 μm of field oxide. Typical confocal micrographs of Vero cells incubated on substrates having isolated tungsten lines with widths of 0.18 μm (Figures 5 (c), (d), and (e)), 2 μm (Figures 5 (f) and (g)), and 10 μm (Figures 5 (h) and (i)) are shown. The cell nuclei (blue), F-actin (red), and tungsten lines (gray) appear against the black silicon oxide background. Both non-dividing (Figures 5 (c), (f), (h), and (i)) and dividing cells (Figures 5 (d), (e), (f), and (g)) were observed in the images.

The 0.18 μm tungsten lines shown in Figure 5 are at least 94 times smaller than the average dimension of the Vero cell nuclei measured on the bare silicon substrate. Unlike Vero cells incubated on bare silicon substrates (Figures 5(a) and (b)), polished silicon oxide, or polished tungsten field area (Figure S2), the cells deposited on the surfaces with isolated tungsten lines elongated into long thin threads. The cell width was largest near the nucleus and resembled an elongated diamond. Cells stretched significantly along the tungsten line, but remained for the most part wider than the 0.18 μm tungsten lines. It is important to note that the tungsten/silicon oxide interface was smooth and continuous, as seen in the cross-section micrographs of Figures 4 and S1. This self-induced cell alignment may be driven by preferential cell adhesion on the tungsten metal region. The contact angle of the cell culture medium (DMEM/F12 + 10% (v/v) FBS) on bare silicon substrate (with native thin layer of silicon oxide at the surface) and tungsten/silicon oxide patterned surface was ~50.0° and ~29.7°, respectively. These results suggest that the patterned tungsten structures increased the hydrophilic characteristic of the surface. In addition to the alteration of the overall cell shapes, cell nuclear geometries also appeared to be affected. The nuclei of non-dividing cells, as shown in Figure 5(c), retained their elongated elliptical shape with the lengths of the long and short axes approximately 21 μm and 7 μm, respectively. However, the ratio between these two axes (~3) was larger than those of cells adhered on the bare silicon (~2.1), polished field tungsten (~1.8), or polished field silicon oxide (~1.8). This indicates a gross deformation in the cell nucleus.

Severely elongated cells also appeared able to perform mitosis. Low- and high-magnification micrographs, shown in Figures 5(d) and 5(e), respectively, depict a dividing cell, likely in anaphase. Threads of condensed chromosomes are being pulled in a direction approximately perpendicular to the metal line axis. The presence of mitosis in the cells is an indication that the cells were alive and that the gross cell deformations did not suppress their division. These results also show that cell geometries can be altered on flat tungsten/silicon oxide nano-composite surfaces without external mechanical assistance. Remarkably, the dimensions of the patterned structures needed to generate these cell morphology changes were significantly smaller than the dimensions of the cell: the width of the patterned structure is only ~1.1% of the average width of the cell nucleus. This demonstrated that cells have high affinity to adhere to the tungsten regions/pattern.

Similar behavior was observed for cells encountering an isolated 2.0 μm wide tungsten line. Three individual Vero cells are shown in Figure 5(f) attaching on a single thin tungsten line. A dividing cell (likely in metaphase), located at the center of the micrograph and highlighted with a square box, is further magnified in Figure 5(g). It is interesting to note that the widths of non-dividing cell nuclei shown in Figure 5(f) were similar to those observed for cells deposited on the surfaces with the 0.18 μm tungsten lines (Figure 5(c)). This suggests that the cell nuclei may have a limit on their ability to deform. Such a limitation would likely be determined by the maximum amount of force generated by the F-actin filaments and the compliance of the cell nuclei; i.e. stiffer nuclei would require larger amounts of actin-force to deform.

Vero cells deposited on a surface with isolated 10 μm tungsten lines fit readily within the 10 μm line, in contrast with the results observed for the 0.18 and 2.0 μm tungsten lines, in which a significant fraction of the cell ‘spilled over’ the edge of the thin tungsten lines. To fit the entire cell body within the 10 μm metal line, the Vero cells shown in Figure 5(h) elongated and their lateral dimension decreased. The elliptical-shaped nuclei had widths approximately the same as the tungsten line (~10 μm), while the length of the nuclei was ~26 μm. The ratios between the long and short axes of these cells were ~2.6.

### 3.4. Kinetics of cell adhesion to tungsten patterns

**Figure 6.**
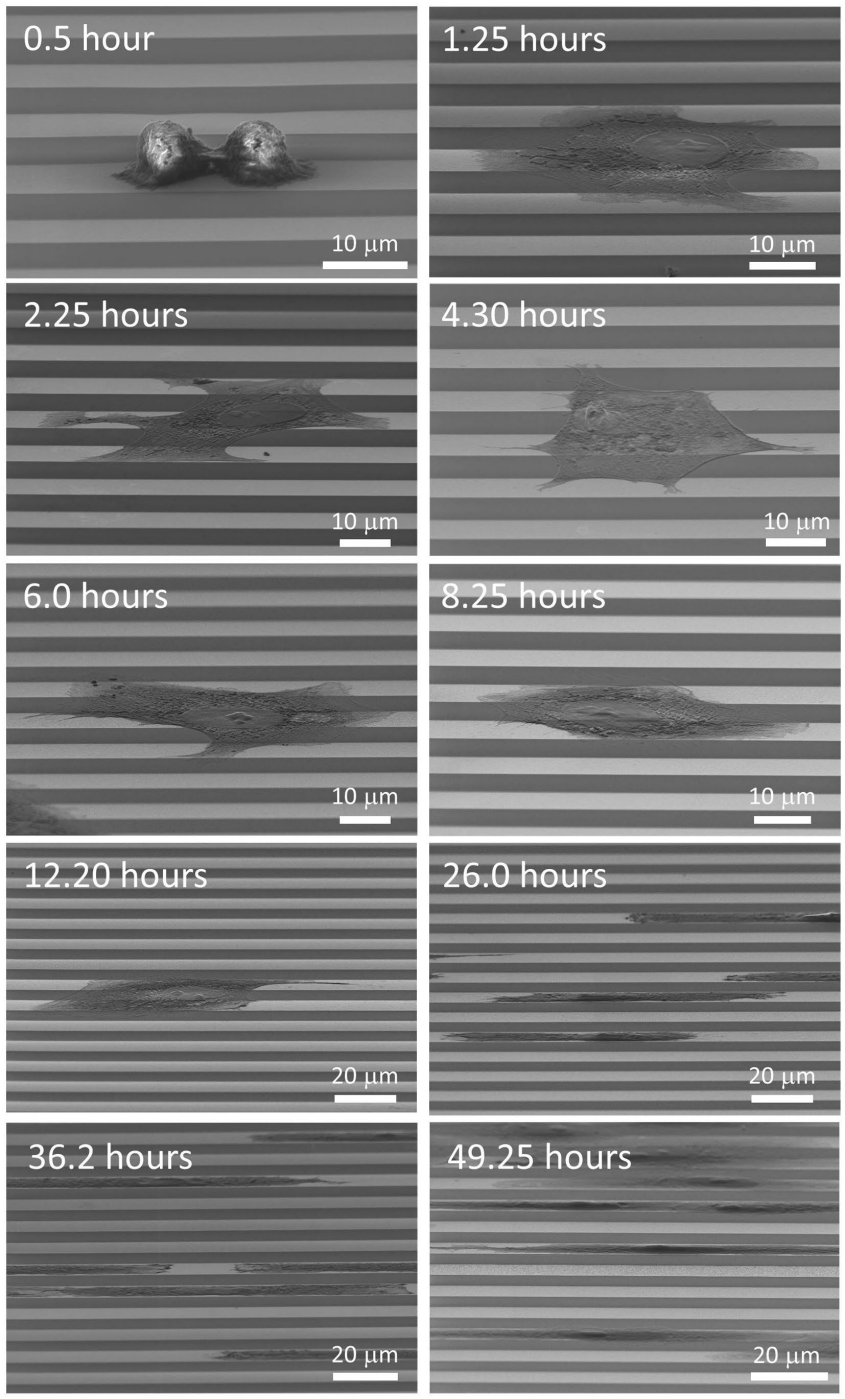
SEM micrographs (70^o^ tilted) of Vero cells on 10 μm comb structures with incubation time between 0.5 and 49.25 hours.

To further understand cell attachment and alignment behavior on the 10 μm comb structures, a time course study was conducted with cells incubated for 0.5, 1.25, 2.25, 4.3, 6, 8.25, 12.2, 26, 36.2, and 49.25 hours. The comb structures consisted of alternating 10 μm wide metal and silicon oxide parallel lines. Cells were seeded at a concentration of 0.5 × 10^5^ cells/mL. Cell morphology was characterized for each timepoint. Representative 70^o^ tilted SEM micrographs of adherent cells after various incubation time are shown in Figure 6. These images show how cell morphology changes between the initial seeding and the 49.25 hour timepoint. Initially, cells attached themselves onto the surface and retained their rounded shape after 30 minutes of incubation with short cytoplasmic projections extending on the surfaces. After one hour of incubation, cells spread in all directions covering both tungsten and silicon oxide regions. Cell diameters are in the order of tens of microns. In most cases, cells adhered to three or more metal lines. Careful inspections of these cells show lamellipodia and filopodia preferentially extended along the tungsten lines. This behavior becomes more obvious with time. After 8 hours of incubation, some cells began to retract in a direction perpendicular to the line axes while cytoplasmic projections extended further along the tungsten lines. The morphological changes continued beyond 26 hours until the cells were only in contact with one or two tungsten lines. Over the period of observation, cells also increased in number in the areas observed indicating cells were able to proliferate on the devices.

### 3.5. Possible driving forces for preferential cell adhesion

Three material properties that may play a role in the observed phenomena include: surface energy, surface roughness, and the ability of the surface to adsorb protein, the latter of which may also be influenced by the former. Hallab et al. [62] and Gentleman and Gentleman [63] suggested that cell attachment and cellular adhesion strengths on metals generally increase with surface energies. Tungsten has one of the highest surface energies of any elemental metals (~3.3 J/m^2^) [49] and is approximately 12 times larger than the silicon oxide (0.259 ± 0.003 J/m^2^) [51]. Another probable driving force for the preferential cell adhesion on polished tungsten structures is their surface roughness (R_rms_) which is ~1.7 times greater than the polished silicon oxide (~0.44 nm vs ~0.26 nm). Literature reports [63–68] have indicated that the adhesion of cells can be greatly enhanced by using materials of increasing surface roughness in metals, such as platinum, tantalum, titanium, and titanium alloys. Rough surfaces bind a larger amount of protein and can affect protein conformation. The ability to preferentially adsorb proteins can in turn lead to favorable cell attachment.

**Figure 7.**
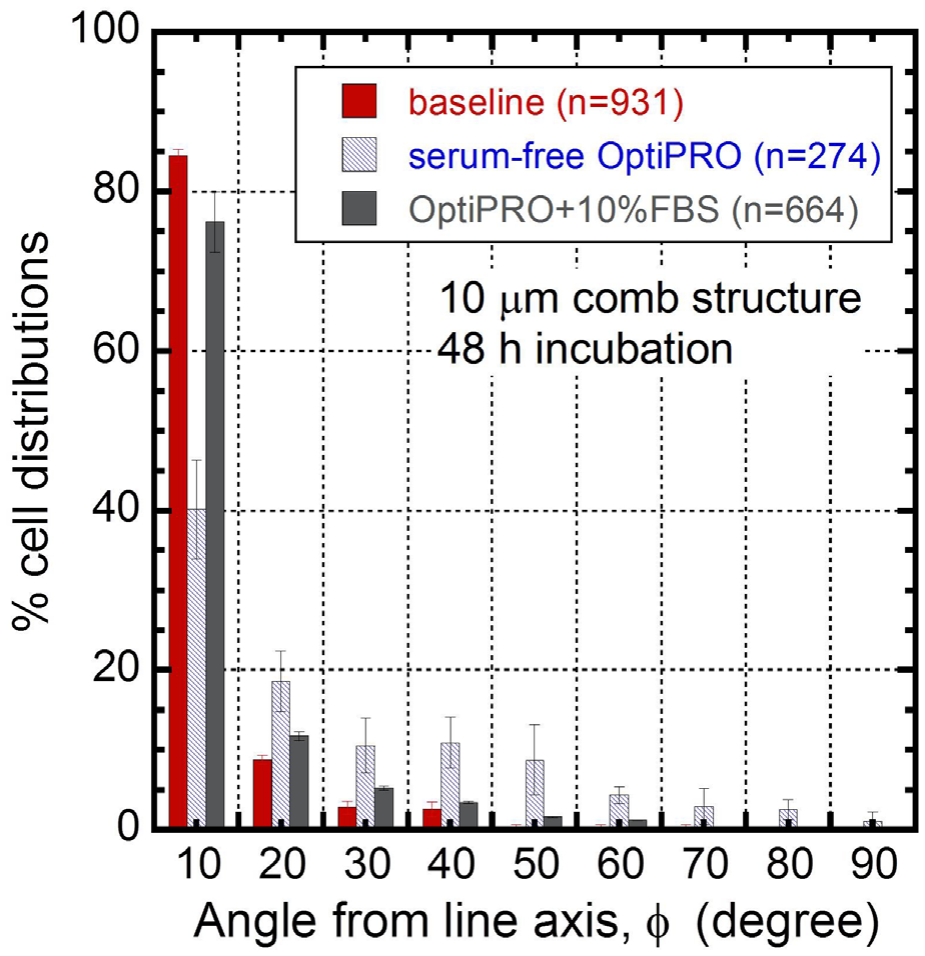
Cell orientation distributions on 10 μm comb structures when cultured in baseline (containing 10% FBS), OptiPRO (serum free), and OptiPRO + 10% FBS media. The number of cells inspected (n) on the specimens are displayed in the legend.

The culture media can play an important role in this regard by providing the adsorbed proteins that enhance localized cell adhesion [69]. To demonstrate the influences of media composition on Vero cell adhesion on 10 μm comb structures, cells were incubated for 48 hours in three different media as described in section 2.3: (i) baseline medium with 10% FBS; (ii) ultra-low protein Gibco^®^ OptiPRO^TM^ SFM cell culture medium; and (iii) OptiPRO^TM^ with 10% FBS. All media were seeded at 0.5 × 10^5^ cells/mL. The distributions of the cellular orientations and the total number of cells on the 10 μm comb structures can be seen in Figure 7. Each bar in the figure represents the portion of the cell population having nuclei that fall within 10° of the line axis. For example, the second bin corresponds to the total distribution of cells that are aligned in two angular ranges from the metal axis, from 10° to 20° and -10° to -20°. For specimens with perfectly randomized cell nuclei orientations, each interval should contain 11.1% of the cell population. Error bars correspond to one standard deviation from three groups of measurements. The figure shows that ~85 ± 1% of cells were oriented within ± 10^o^ of the line axes when the baseline media was used. In contrast, only ~40 ± 6% of the cell population were oriented in the same angular interval in OptiPRO^TM^ media. When 10% FBS was added to OptiPRO^TM^ media, the cell alignment improved to ~76 ± 4%. Results demonstrate that FBS enabled cell alignment on the W-CMP devices. Similar behavior has also been reported by Teixeira et al. [69] where they observed lower cell alignment performance in the absence of FBS in culture medium.

### 3.6. Effects of comb structure patterns on the adherent cell morphology

**Figure 8.**
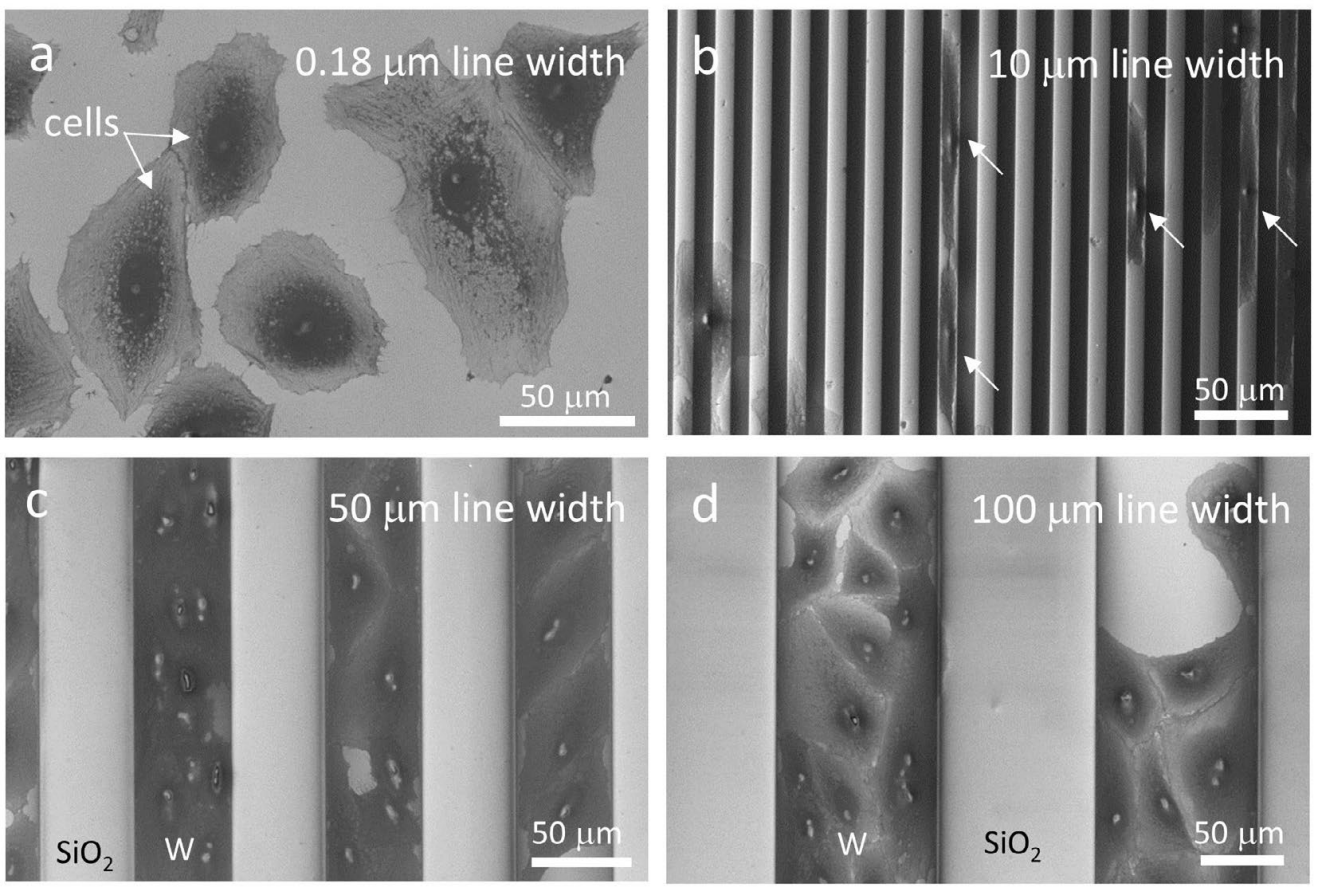
SEM micrographs revealing changes in cell adhesion characteristics with tungsten line widths of (a) 0.18 μm, (b) 10 μm, (c) 50 μm, and (d) 100 μm. Tungsten line width and silicon oxide spacing in the comb structure are identical in each image.

The influences of comb structure patterns on Vero cell morphologies were investigated. These comb structures consisted of alternating metal and silicon oxide parallel lines of the same width. Representative high-resolution SEM micrographs of cells incubated on four different groups of comb structures with line widths of 0.18, 10, 50, and 100 μm are displayed in Figures 8(a)–(d), respectively. The width and the sampling areas of each patterned structure were at least 1 mm and 1.8 mm^2^, respectively. Since the widths of tungsten and silicon oxide lines were identical and alternating, they each cover approximately 50% of the comb structure. Vero cells were incubated for 24 hours on these comb structures in culture media with an initial cell concentration of 2.0 x 10^5^ cells/mL.

A typical micrograph of Vero cells on a 0.18 μm wide line comb structure is shown in Figure 8(a). Although the lines are indiscernible because of the magnification, the lines were aligned vertically in the image. Cells on the 0.18 μm comb pattern did not display the same elongation as observed with the isolated 0.18 μm tungsten lines, demonstrating that the response to the tungsten metal were pattern-dependent. With the 10 μm comb pattern, cells once again preferentially attached to the tungsten lines (light gray vertical lines); however, unlike on the 0.18 μm comb structure, the cells behaved more similarly to the isolated line patterns seen in Figure 5. In many instances, cells remained entirely within the tungsten lines without any contact with the silicon oxide surface (highlighted with solid arrows in Figure 8(b)). Furthermore, unlike the arbitrary orientations of cells on the 0.18 μm comb structure, a large portion of the cells on the 10 μm comb structure aligned themselves in a direction parallel to the tungsten lines.

As the patterned line width increased to 50 μm, the majority of the Vero cells adhered to the tungsten lines, as seen in Figure 8(c). This figure reveals a representative SEM micrograph of cells cramped on tungsten metal lines, while no cell is observed in the silicon oxide region. Out of 378 cells inspected in this comb structure, only seven of them (~1.9% of the sampled population) adhered to silicon oxide, while the rest (~98.1%) were attached to the tungsten lines. This preferential behavior is also observed in the large 100 μm wide parallel line patterns (Figure 8(d)). Of the 1434 cells inspected, only three cells were found to have adhered to the silicon oxide region, representing a 99.8% attachment affinity for tungsten. The cells on the 100 μm wide lines oriented themselves in a more random fashion compared to those on 10 μm and 50 μm comb patterns. Additional fluorescence confocal micrographs of cells adhered on patterned structures are presented in Figure S4.

### 3.7. Extracting cellular parameters to quantify the affinity of cells for tungsten

**Figure 9.**
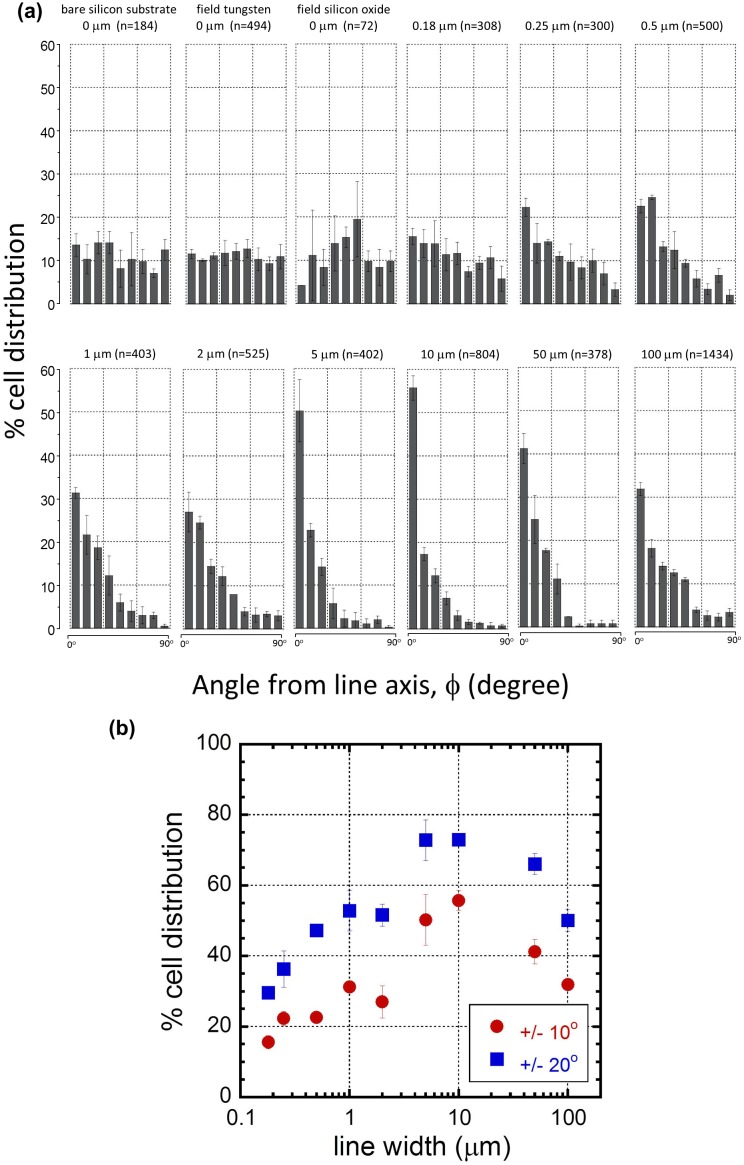
(a) Plots of percent cell distribution as a function of the angle between the nuclei long axis and the metal line axis. They include results from bare silicon substrate, polished field tungsten, polished field silicon oxide, and comb structures with line widths in the range of 0.18 μm to 100 μm. The number of cells inspected in each comb structures (n) is included in each chart. Each bar represents a 10° bin of deviations from the line axis (either +/−), i.e. a cell with a nucleus major axis of −24^o^ would be counted in the second bin from the left in each plot. Error bars correspond to one standard deviation of three independent cell populations. The initial cell concentration of the culture media was 2 ×10^5^ cells/mL. (b) Plot of cell orientation distributions as a function line width.

To quantify the preferential adhesion behavior, cell orientation distributions for different comb structures were plotted in Figure 9(a). Unless otherwise stated, all data spreads and error bars presented are equal to one standard deviation calculated from three independent groups of cells. More than 6500 cells were manually characterized. The total number of adherent cells in each comb structure (n) is labeled on each plot. Figure 9(a) indicates nuclei on bare silicon substrates, polished field tungsten, and polished silicon oxide did not exhibit any distinct preferential orientation. The nuclei orientation distribution on the 0.18 μm comb structure, shown in Figure 9(a), is similar to that of bare silicon, polished tungsten, and silicon oxide surfaces, i.e. there is no preferred alignment. As line widths of the comb structures increase from 0.25 μm to 10 μm, the fraction of cell nuclei oriented within ±10° of the metal line axis increased from 22% (0.25 μm comb structure) to 56% (10 μm comb structure). For comb structures with line widths above 10 μm, the cell orientations started to return to a more uniform distribution, i.e. they became increasingly randomized. This is further illustrated in Figure 9(b), in which the percentage of cell nuclei aligned within ±10° and ±20° of the metal line axis was plotted against the comb structure line widths. Similar line width-dependence of cell orientations was observed by Nakamoto et al. [7] on micropatterned cell-adhesive polystyrene topographic structures. They showed that all human mesenchymal stem cells deposited on the 20 μm line patterns did align along the line axes, but their orientations were increasingly randomized on the wider lines.

**Figure 10.**
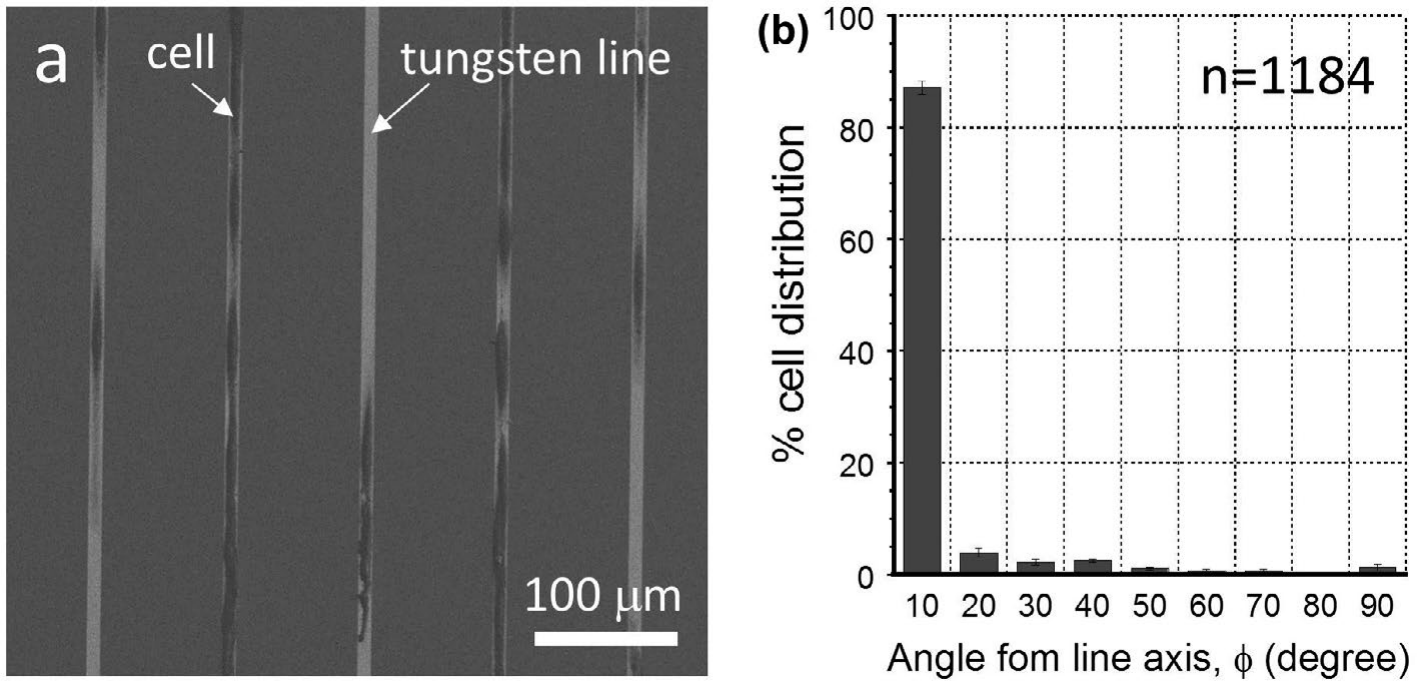
(a) Representative SEM micrographs of a comb structure with 10 μm wide tungsten lines and 90 μm spacing. (b) Plot of percentage cell distribution as a function of angles between the major axis of the nuclei and the metal axis. Parameter n is the total number of cells sampled.

Micrographs and cell nuclei orientation results clearly show the influence of line width on cell nuclei alignment; however, the results were from patterns that had identical tungsten and silicon oxide widths. To demonstrate the effect of spacing between tungsten lines on cell alignment, additional experiments were performed on a comb structure with 10 μm wide tungsten lines separated by 90 μm of silicon oxide. The physical dimensions of this structure are described in Table 1. Representative SEM micrographs of cells adhered on this structure are displayed in Figure 10(a). A total of 1184 cells were observed and used to generate data on cell behavior. The majority of the cells attached on the tungsten lines, while only a few attached on the silicon oxide region. A significant portion of the cells aligned parallel to the metal line axis. Quantitative cell nuclei orientation results collected from this comb structure are plotted in Figure 10(b). The figure shows 87 ± 1% of cells were aligned within the first 10° interval from the metal line axis. The cell alignment was significantly more efficient than what was observed for the 10 μm tungsten/10 μm silicon oxide comb structure, which had only 56% of the cell population in the same angular interval. These results demonstrate that the spacing between the metal lines is an important geometric parameter that controls cell alignment. A typical confocal micrograph of these well-aligned cell structures is displayed in Figure S4(e).

### 3.8. Modeling cell-tungsten coverage-dependence on comb structure line widths

**Figure 11.**
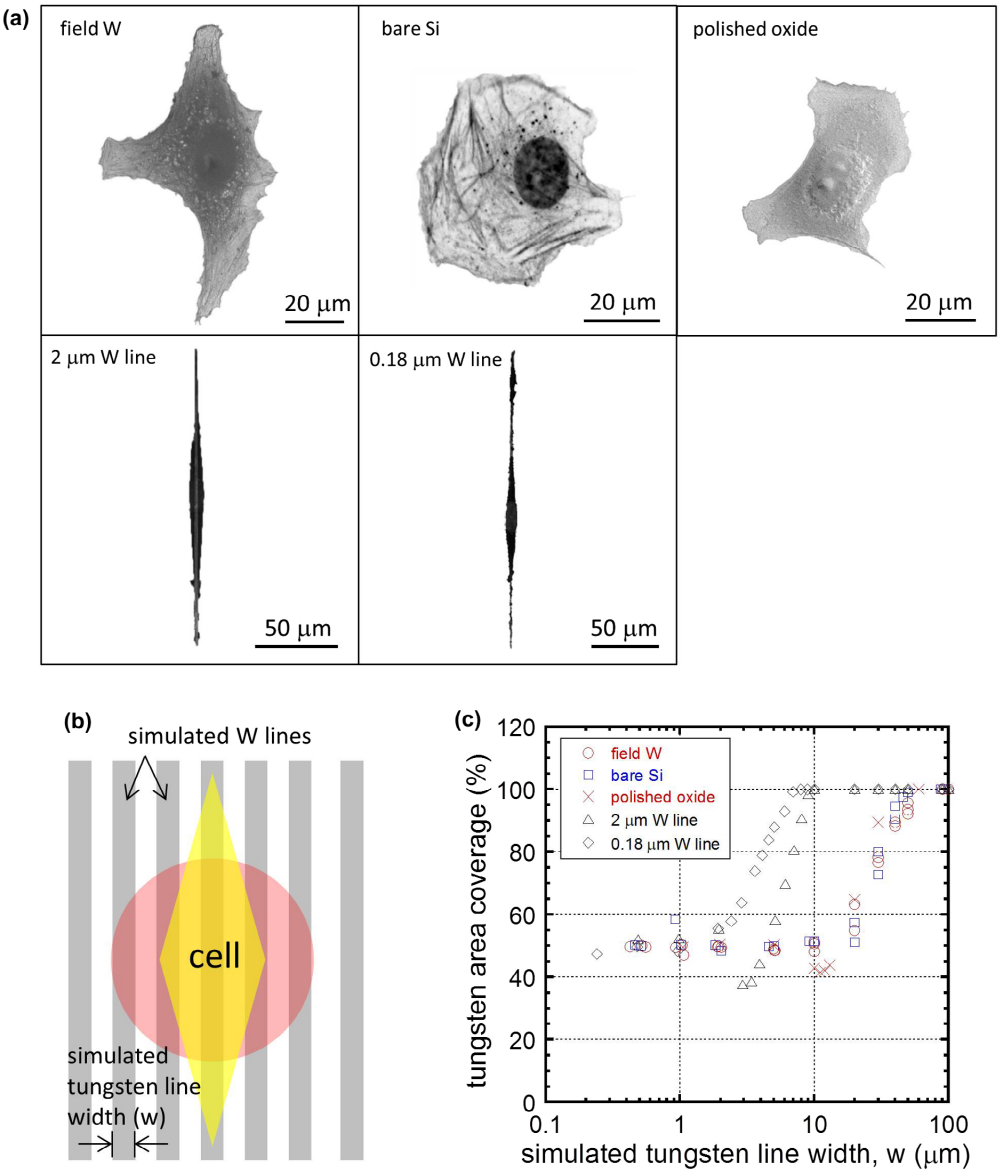
(a) Observed cell morphologies on different surfaces (polished field tungsten area – field W; bare silicon – bare Si; polished silicon oxide – polish oxide; surface with isolated 2 μm line – 2 μm W line; surface with 0.18 μm tungsten isolated line – 0.18 μm W line) with a superimposed simulated comb pattern used in the calculation of coverage in (c). (b) Schematic drawing indicating cell orientation relative to the simulated comb structure; the pink circle and yellow diamond represent cells with random and elongated shapes, respectively. (c) Cell-tungsten coverage of cells plotted as a function of simulated tungsten line widths (w).

The micrographs shown in Figure 8 and the cell-tungsten coverage results plotted in Figure 9 reveal a strong correlation between comb structure line widths and cell deformation characteristics. Cells exhibited the strongest elongation and alignment characteristics when the comb structure line widths were near 10 μm. Such behavior appears to be motivated by the propensity of cells to maximize their contact area with tungsten. To quantitatively demonstrate this cell geometry-dependent behavior, the cells’ coverage of tungsten, in terms of area, was calculated as a function of simulated comb structure metal line widths (w), an example of which is given in Figure 11. Cell geometries used in this part of the analysis were obtained from actual adherent cells on various surface structures (see Figures 11(a) and S5). The geometries represent the shape of cells observed on a polished field tungsten area, bare silicon substrate, polished silicon oxide, and on isolated 2 μm and 0.18 μm tungsten lines. Figure 11(a) shows the cell incubated on a polished field tungsten surface was irregularly shaped and had approximate dimensions of ~97 μm wide and ~107 μm long. Randomly shaped cells on bare silicon had approximate dimensions of ~56 μm x ~58 μm. Cells on polished oxide had an irregular shape with a length and width of 54 μm and 48 μm respectively. The elongated cell on the isolated 2 μm tungsten line was ~179 μm long and ~11 μm wide, whereas the cell on the 0.18 μm line was ~228 μm long and ~9 μm wide.

Simulated parallel tungsten line comb structures were overlaid on these cell geometries to determine the relationship between the metal line width and percentage of the cell in contact with tungsten, as illustrated in Figure 11(b). In the schematic, the pink circle and yellow diamond represent the variety of cell shapes observed. Cell orientations relative to the comb line structure used in the simulation were as depicted in Figure 11(b). Note that when half of the cell area was in contact with these simulated tungsten lines, the percent cell-tungsten coverage was considered to be 50%. Likewise, when the entire cell was in contact with the tungsten surface, the coverage was considered to be 100%.

Results plotted in Figure 11(c) show that when irregularly shaped cells (Figure 11(a) top), such as those observed on the polished field tungsten area, bare silicon substrate, and polished silicon oxide, were placed on the simulated comb structures with tungsten line widths smaller than 10 μm, the cell-tungsten coverages were ~50%. Increasing simulated line widths beyond 20 μm results in larger tungsten coverage for these irregularly shaped cells. Tungsten coverage reaches 100% at simulated metal line widths larger than 100 μm, at which point the entire cell is in contact with tungsten.

Cells with elongated geometries (see Figure 11(a) bottom) have tungsten coverage of ~50% when simulated tungsten line widths are smaller than 2 μm, which is similar to the coverage observed for irregularly shaped cells. This suggests that there is no substantial benefit for cells to deform and elongate on fine-line comb structures because it does not increase the cell-tungsten contact area. However, as the comb structure line width increases to ~6 μm, the tungsten area coverage of elongated cells increases to above 70%, which is noticeably higher than those of the irregular shaped cells. There is therefore a window between ~2 and 50 μm, during which the cell deforms to align along the tungsten line, resulting in more extensive cell-tungsten contact (Figure 11(c)). The more extensive coverage that results from elongation, however, starts decreasing at line widths greater than 10 μm, at which point the contact between the irregular shaped cells and tungsten starts increasing beyond 50%.

The simulated tungsten coverage results presented in Figure 11 are consistent with the experimental observations shown in Figure 8 and Figure 9, where cells do not have distinct preferential orientations on the narrow 0.18 μm line comb structure, even though cells on the isolated line with the same width elongate into thin threads (see Figure 5). These simulation results also appear to match with the experimental findings demonstrating that a maximum number of cells align parallel to the metal lines at widths of ~10 μm. Beyond this characteristic line width, the benefit for cells to maintain an elongated shape is reduced with wider lines. The simulated results are also consistent with the observations shown in Figure 9, where cells on 50 μm and 100 μm lines have weaker preferential orientation compared to those on 10 μm lines.

### 3.9. Cell alignment performance of mechanically reworked integrated circuits

**Figure 12.**
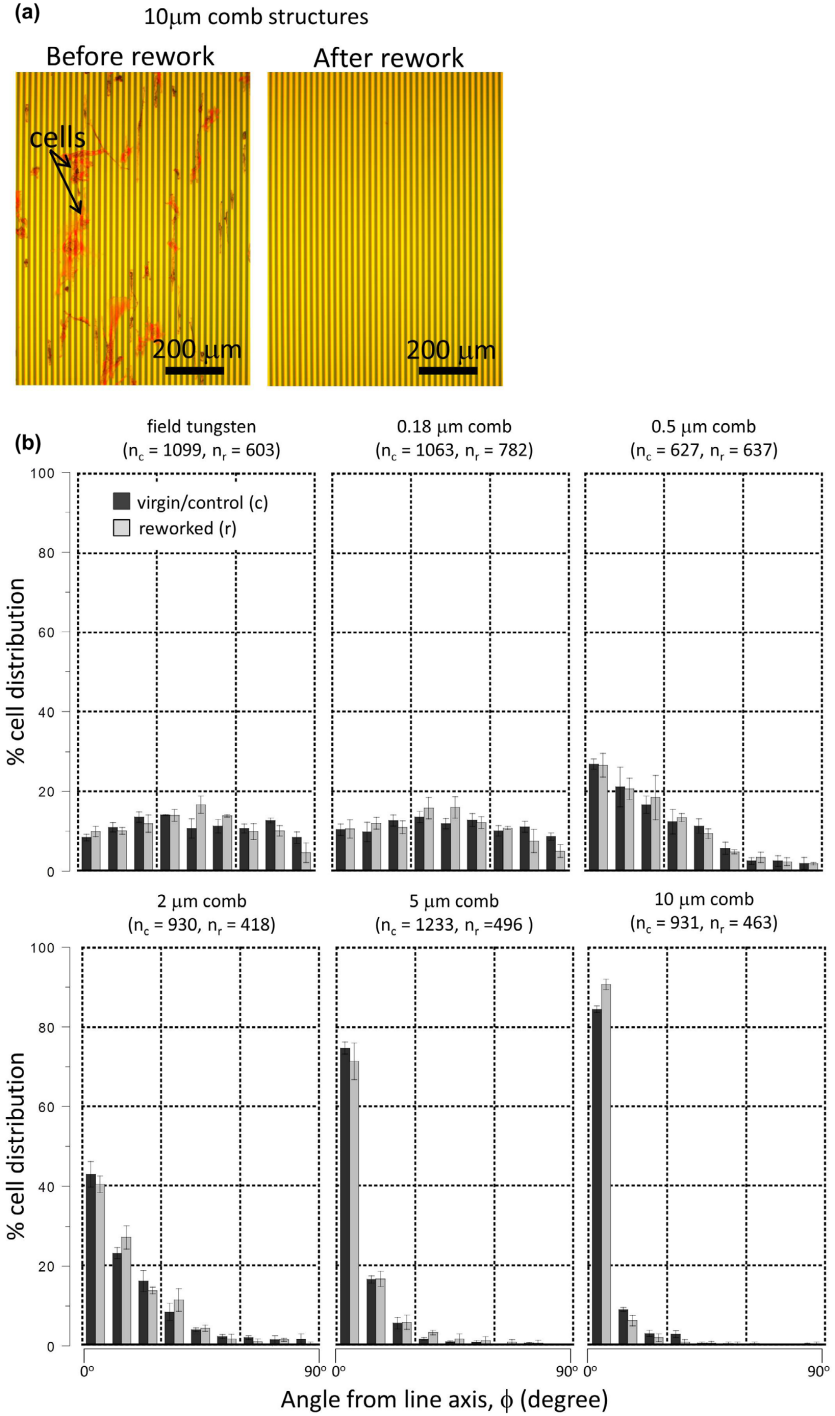
(a) Typical optical micrographs of the same 10 μm comb structures before and after the rework process to remove QT-35 cells. (b) Adherent cell orientations on virgin/control (c) and reworked (r) specimens. n_c_ and n_r_ represent the numbers of cells characterized within each comb structure on virgin and reworked specimens. Error bars correspond to one standard deviation. Initial cell concentration of the culture media is 0.5 × 10^5^ cells/mL.

Smooth and flat W-CMP ICs fabricated for this work were constructed with mechanically robust tungsten and silicon oxide. With these materials, it is possible to remove particles, residues, or other surface contaminants using a chemical-free mechanical scrub without damaging the patterned structures. This is common practice in the semi-conductor fabrication industry to improve production yield, and will provide a unique advantage to these IC-based cell alignment devices [53,54]. To demonstrate this capability, QT-35 cells were used as controlled contaminants, and the mechanical rework processes discussed in section 2.4 were performed. Figure 12(a) shows typical optical micrographs of a 10 μm wide comb structure before and after mechanical scrubbing. Unstained QT-35 adherent cells are in red as the result of optical light diffraction. Additional representative micrographs collected from other comb structures are displayed in Figure S6. It is obvious that all QT-35 adherent cells on these surfaces were successfully removed. No visible scratches or residues were observed on these reworked surfaces. Tungsten line patterns were not distorted or damaged by mechanical scrubbing.

Vero cells were seeded on the virgin control and reworked specimens according to the methods described in section 2.4 at a cell concentration of 0.5 × 10^5^ cells/mL. They were incubated for 48 hours. Cell nuclei orientations on field tungsten and comb structures (with 0.18, 0.5, 2, 5, and 10 μm tungsten line widths) were plotted in Figure 12(b). There were over 9000 Vero cells manually characterized. The number of cells within the comb structures on the virgin control (n_c_) and reworked (n_r_) samples for each comb structure were included in this figure. Results show that the cells were randomly oriented on the tungsten field area and the 0.18 μm comb structure surfaces. As the line widths increased from 0.5 μm to 10 μm, the percentage of cells oriented along the tungsten line increased (Figure 12(b)). For comb structures with line widths of 10 μm, the fraction of cell nuclei oriented within 20° of the metal line were ~93 ± 1% and ~97 ± 1%, respectively. This demonstrates that the mechanical scrubbing process did not degrade the effectiveness of cell alignments on the 10 μm comb structures. Close inspections of cell orientation results in other comb structures confirm the mechanical robustness of the surfaces, which maintain their cell alignment efficiency. This is believed to be the first report in the literature demonstrating that a cell alignment device can be reworked using a simple chemical-free mechanical scrubbing technique without degrading its functionalities. Such rework-ready functionality allows enhanced production yield during large-scale manufacturing because it uses an inexpensive mechanical scrubbing technique, and enables reuse by end-users [53,54].

### 3.10. Alignment performance

The Vero cell alignment performances on the 10 μm comb structure are comparable with those reported in the literature [7,32,69–73]. Teixeira et al. [69] examined corneal epithelial cell alignment behaviors on patterned silicon oxide line structures with dimensions of 70 nm wide ridges, 600 nm deep grooves, and 400 nm pitch. Their results show that ~35% of cells can be aligned within 10° of the line axes. Poudel et al. [32] investigated orientation characteristics of human neuroblastoma (cell line SH-SY5Y) cells on micro-patterned 10 μm wide retinoic acid lines. They showed that ~84% of adherent cells oriented within 10° of the line pattern. As a comparison, Figure 12(b) shows that at least ~85 ± 1% of Vero cells were aligned in the same angular interval on W-CMP 10-μm comb structures. Nakamoto et al. [7] studied the orientation of human mesenchymal stem cells on micropatterned cell-adhesive polystyrene strips, i.e. topographical structures with sub-micron scale trench depths. They reported that the best cell alignments were observed on 20 μm wide strips with 100% of adherent cells oriented within 15° of the pattern directions. As for Vero cells on the 10 μm comb structures, ~90 ± 2% of the cell nuclei population oriented within the same angular interval. This analysis shows how W-CMP devices exhibit similar cell alignment performances as some of the best and representative methods reported in the literature. Additionally, the chemical-free mechanical rework capability provided by this technique provides a significant advantage during manufacturing and end-user field usages, with respect to existing devices.

## 4. Conclusions

Vero cells could adhere to tungsten chemical-mechanical polished surfaces with isolated lines and comb structures. Results show cells preferentially attached to tungsten over silicon oxide. Those cells that adhered onto the isolated tungsten lines elongated along the surface of the metal. Micrographs show that cells were still able to divide even though they were grossly deformed on the tungsten metal lines. The line widths within the comb structures affected the cell nuclei orientations. Results show that only ~22% of cell nuclei on 0.18 μm wide line comb structures oriented within 20° of the metal line axis. The aligned nuclei population increased to more than ~93% on the repeated 10 μm tungsten/silicon oxide line comb structures. A simple model was developed to simulate and predict the preferential cell geometries as a function of line width in a comb pattern. The functionalities of these devices were maintained even after the removal of surface contaminants by a simple chemical-free mechanical scrub rework process with de-ionized water.

## Disclosure statement

No potential conflict of interest was reported by the authors.

## Funding

This work was supported by Natural Sciences and Engineering Research Council of Canada [grant number RGPIN-355552].

## Supplemental data

Supplemental data for this article can be accessed here. https://doi.org/10.1080/14686996.2017.1388135

## Supplementary Material

Supplementary_figures_9_18.pptx
