# Supplementary material for: Manipulating mammalian cell morphologies using chemical-mechanical polished integrated circuit chips
Source: Sci Technol Adv Mater. 2017 Oct 27;18(1):839–56. doi: 10.1080/14686996.2017.1388135 (PMC5678500; doi:10.1080/14686996.2017.1388135)
Supplement: Supplementary_figures_9_18.pptx [file TSTA_A_1388135_SM2004.pptx]

## Slide 1
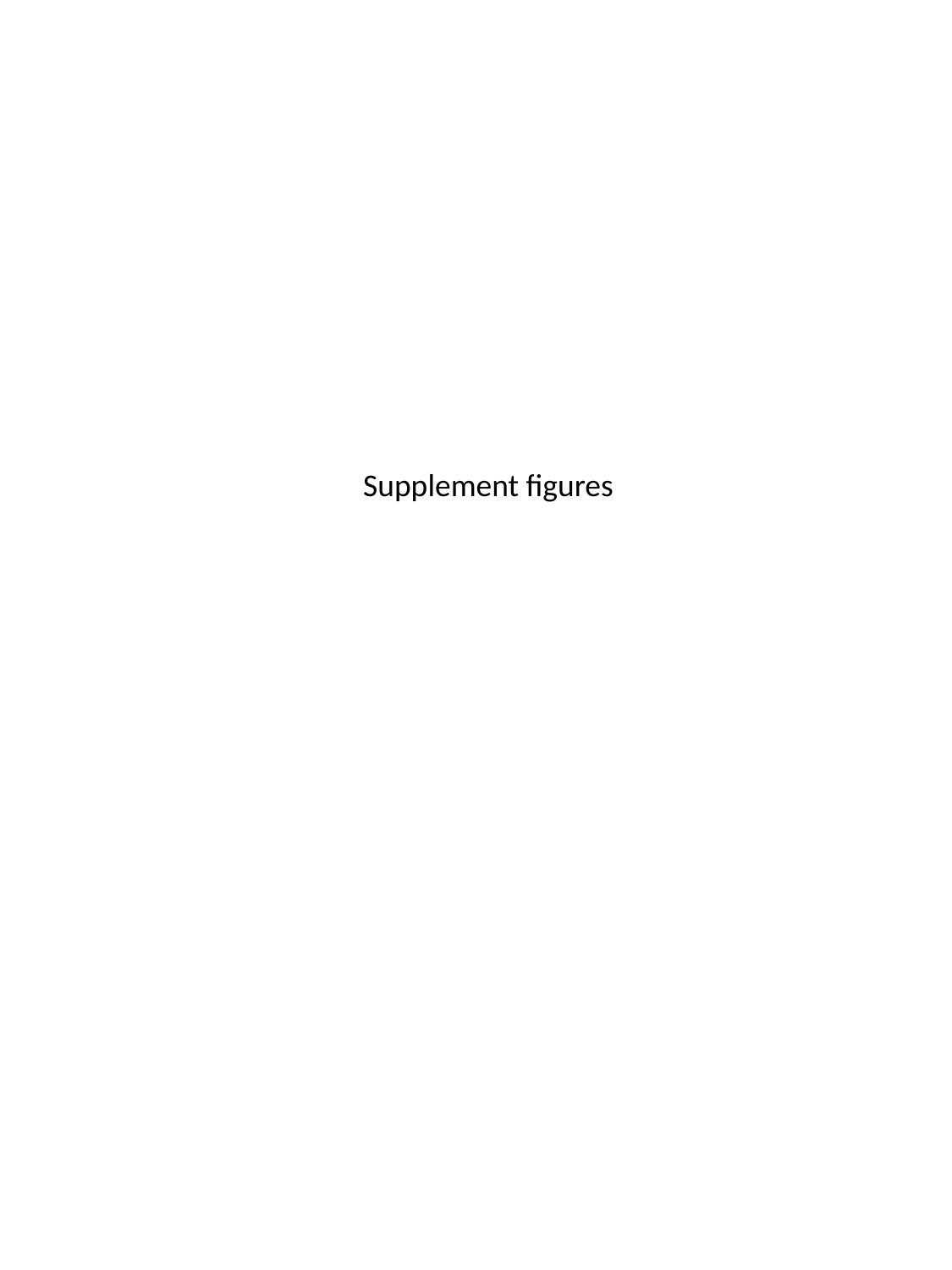

Supplement figures

## Slide 2
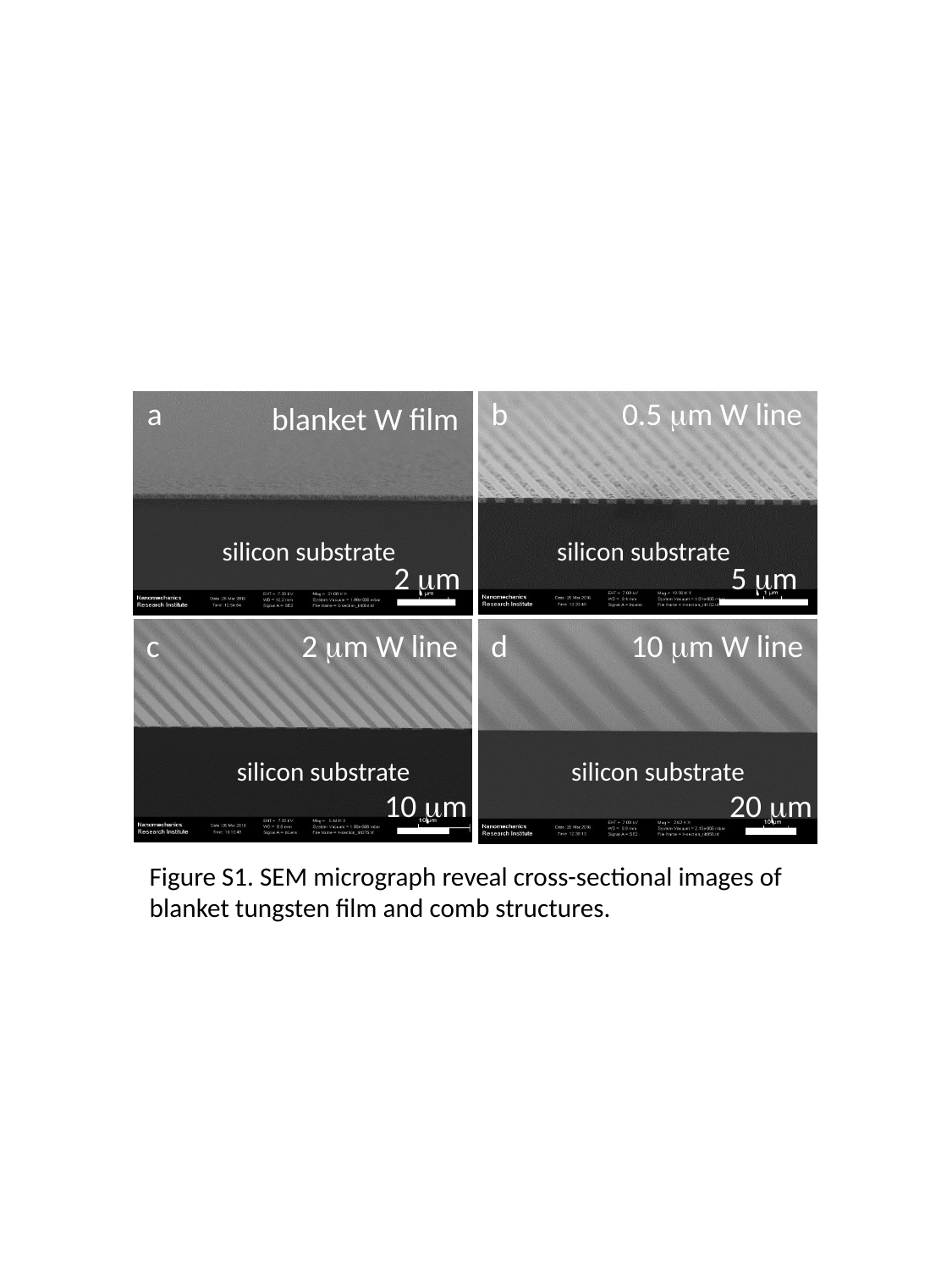

a
b
0.5 mm W line
5 mm
blanket W film
silicon substrate
silicon substrate
2 mm
d
10 mm W line
20 mm
c
2 mm W line
10 mm
silicon substrate
silicon substrate
Figure S1. SEM micrograph reveal cross-sectional images of blanket tungsten film and comb structures.

## Slide 3
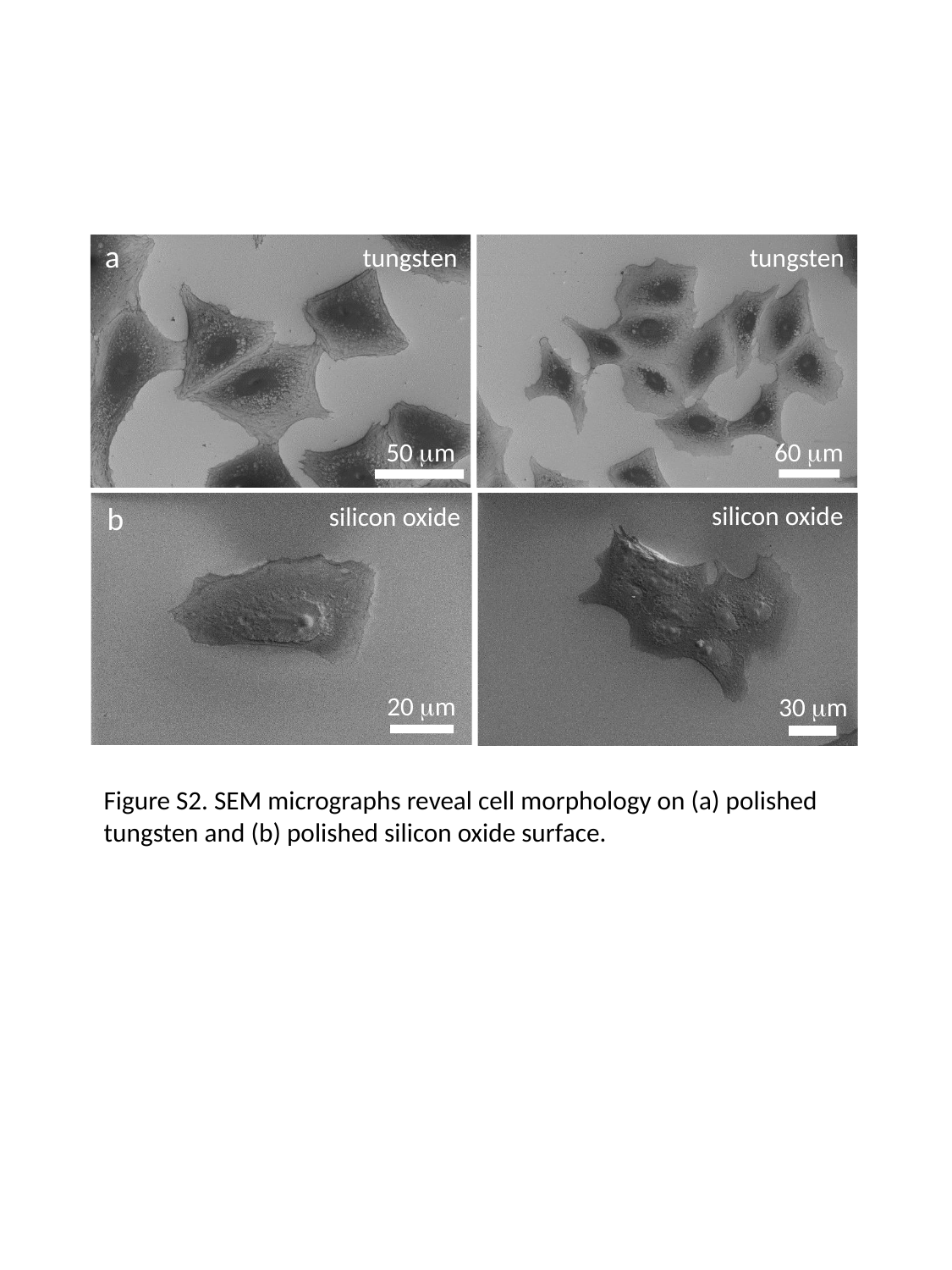

a
tungsten
tungsten
50 mm
60 mm
silicon oxide
b
silicon oxide
20 mm
30 mm
Figure S2. SEM micrographs reveal cell morphology on (a) polished tungsten and (b) polished silicon oxide surface.

## Slide 4
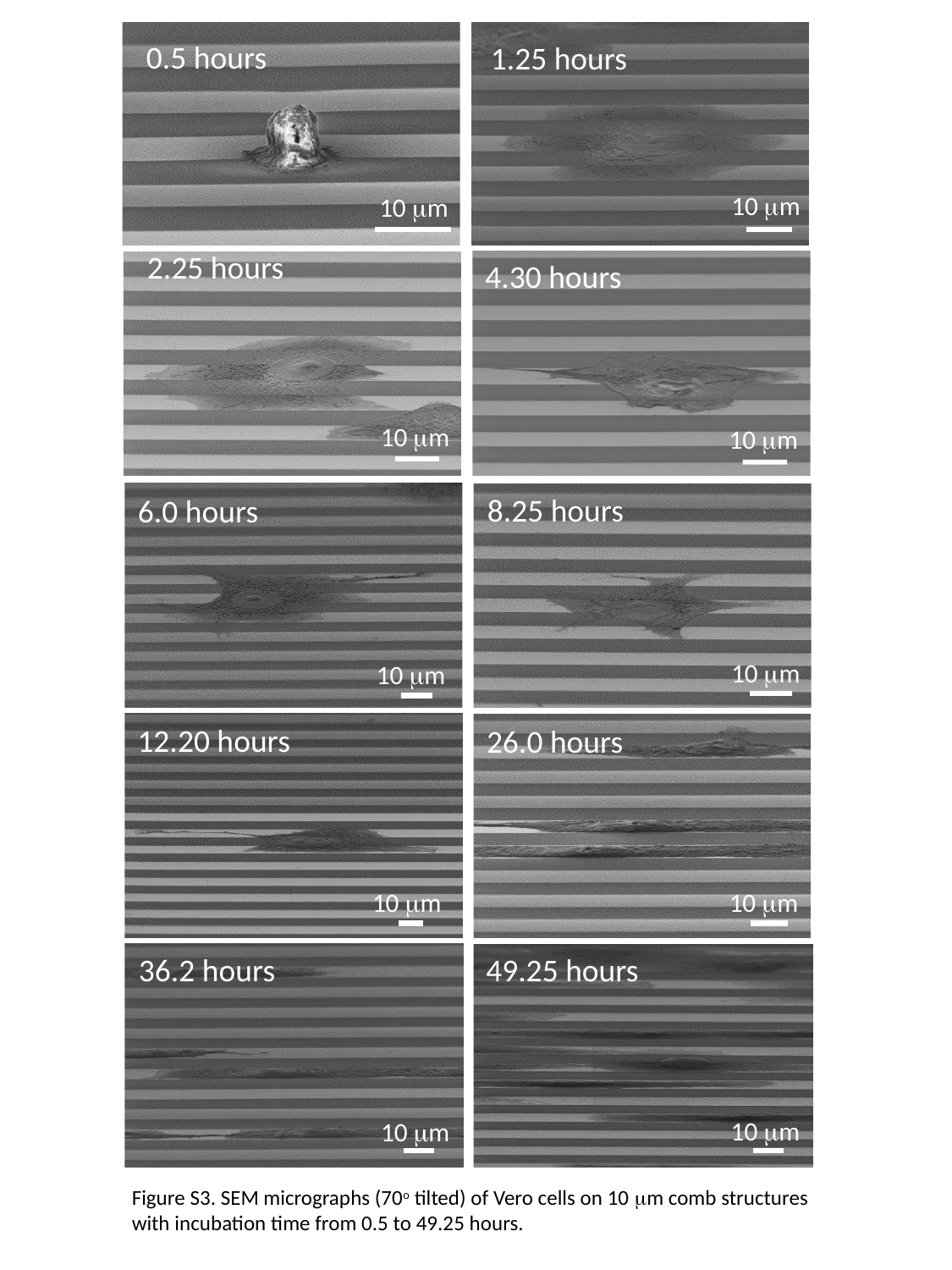

0.5 hours
1.25 hours
10 mm
10 mm
2.25 hours
4.30 hours
10 mm
10 mm
8.25 hours
6.0 hours
10 mm
10 mm
12.20 hours
26.0 hours
10 mm
10 mm
36.2 hours
49.25 hours
10 mm
10 mm
Figure S3. SEM micrographs (70o tilted) of Vero cells on 10 mm comb structures with incubation time from 0.5 to 49.25 hours.

## Slide 5
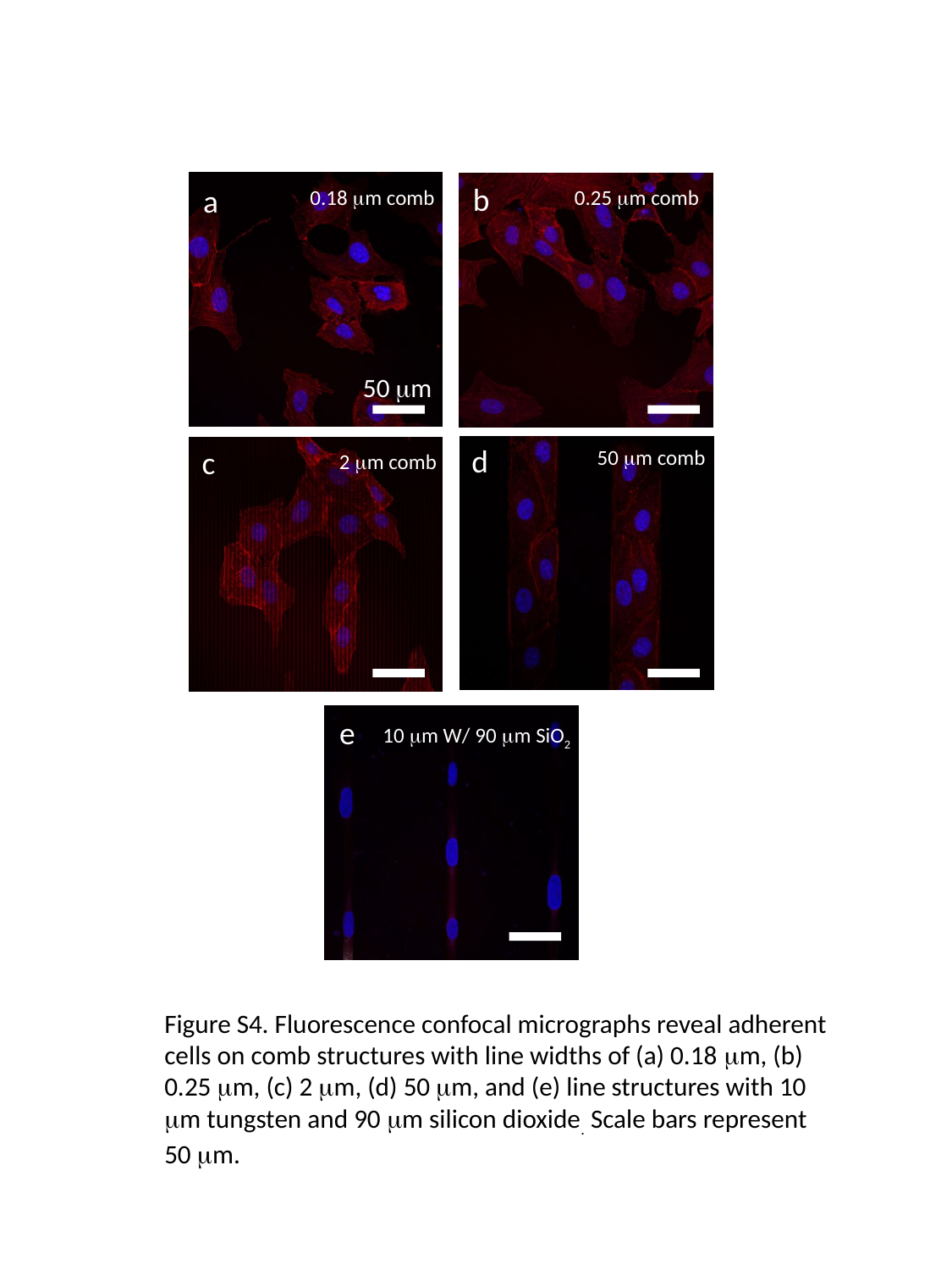

b
a
0.18 mm comb
0.25 mm comb
50 mm
d
c
50 mm comb
2 mm comb
e
10 mm W/ 90 mm SiO2
Figure S4. Fluorescence confocal micrographs reveal adherent cells on comb structures with line widths of (a) 0.18 mm, (b) 0.25 mm, (c) 2 mm, (d) 50 mm, and (e) line structures with 10 mm tungsten and 90 mm silicon dioxide. Scale bars represent 50 mm.

## Slide 6
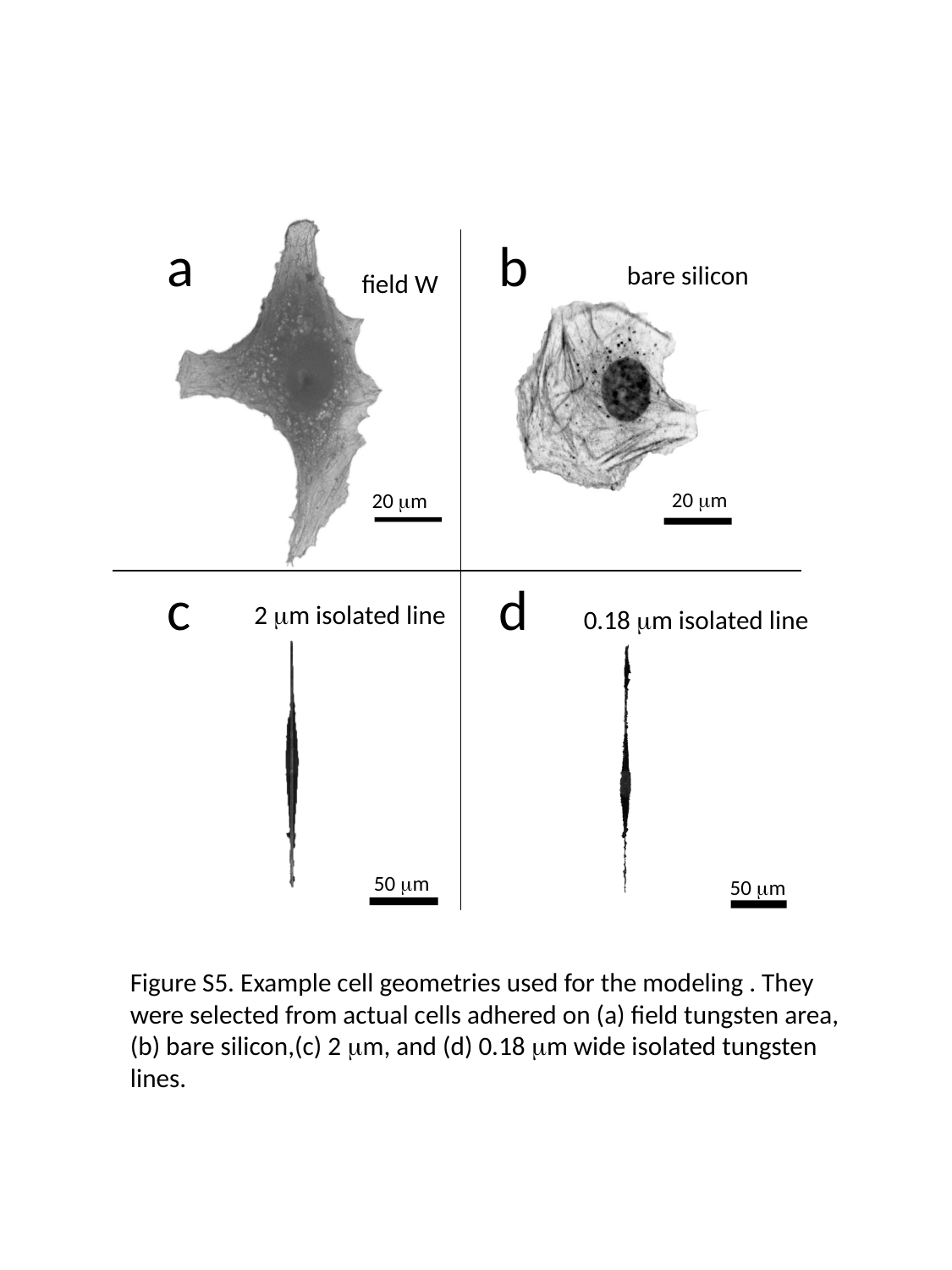

a
b
bare silicon
20 mm
field W
20 mm
c
d
2 mm isolated line
0.18 mm isolated line
50 mm
50 mm
Figure S5. Example cell geometries used for the modeling . They were selected from actual cells adhered on (a) field tungsten area, (b) bare silicon,(c) 2 mm, and (d) 0.18 mm wide isolated tungsten lines.

## Slide 7
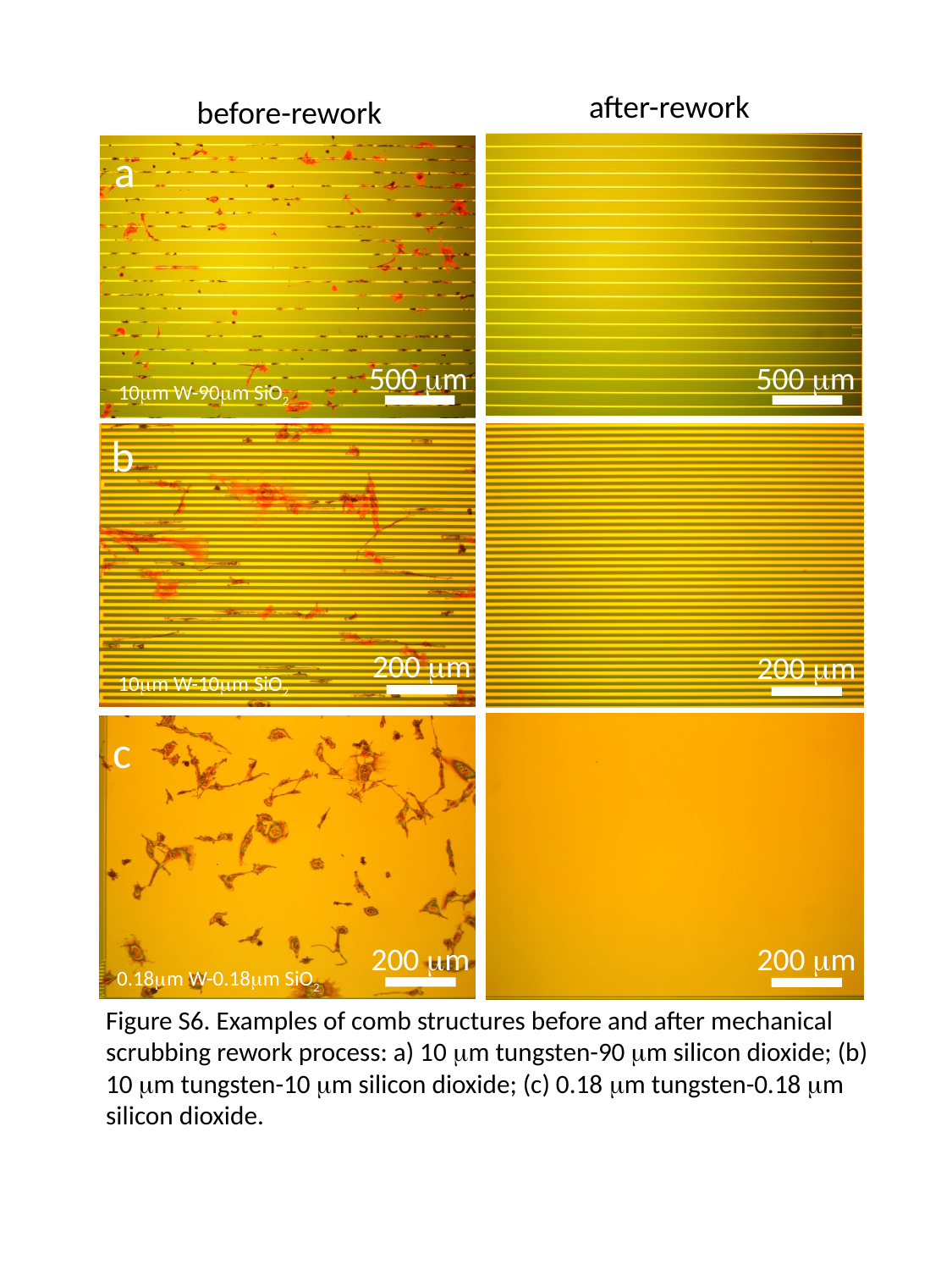

after-rework
before-rework
a
500 mm
500 mm
10mm W-90mm SiO2
b
200 mm
200 mm
10mm W-10mm SiO2
c
200 mm
200 mm
0.18mm W-0.18mm SiO2
Figure S6. Examples of comb structures before and after mechanical scrubbing rework process: a) 10 mm tungsten-90 mm silicon dioxide; (b) 10 mm tungsten-10 mm silicon dioxide; (c) 0.18 mm tungsten-0.18 mm silicon dioxide.
